# Identification of a novel rhoptry protein expressed predominantly in *Plasmodium* sporozoites

**DOI:** 10.3389/fcimb.2025.1749149

**Published:** 2026-01-26

**Authors:** Sunti Oundavong, Takashi Sekine, Motomi Torii, Tatsuhiko Ozawa, Hidetaka Kosako, Naoaki Shinzawa, Tomoko Ishino

**Affiliations:** 1 Department of Parasitology and Tropical Medicine, Graduate School of Medical and Dental Sciences, Institute of Science Tokyo, Tokyo, Japan; 2 Division of Molecular Parasitology, Proteo-Science Center, Ehime University, Toon, Ehime, Japan; 3 Department of Life Sciences and Bioengineering, Laboratory of Molecular and Cellular Biology, Faculty of Engineering, Academic Assembly, University of Toyama, Toyama, Japan; 4 Center for Advanced Antibody Drug Development, University of Toyama, Toyama, Japan; 5 Division of Cell Signaling, Institute of Advanced Medical Sciences, Tokushima University, Tokushima, Japan

**Keywords:** CRISPR/Cas9 system, DiCre system, novel rhoptry protein, *Plasmodium berghei*, proximity-dependent biotinylation, sporozoite

## Abstract

The infective stages of apicomplexan protozoans, such as the malaria parasite *Plasmodium*, possess apical organelles rhoptries and micronemes, which contain secretory proteins required for host cell invasion. The mechanisms mediating invasion are largely conserved among apicomplexan parasites; for example, rhoptry proteins are secreted to form a tight junction prior to invasion, which facilitates parasite entry into target cells. Stage-specific invasion mechanisms have also been described; such as those differentially mediating *Plasmodium* merozoite infection of erythrocytes versus sporozoite stage invasion of mosquito salivary glands and mammalian hepatocytes. Sporozoites are the transmission stage present within the salivary glands of infected mosquitoes, and can efficiently infect the mammalian liver after being deposited in the skin during a blood meal. While some sporozoite rhoptry proteins have been demonstrated to be critical for invasion of mosquito salivary glands and mammalian hepatocytes, their comprehensive molecular mechanisms have not been elucidated due to the limited availability of material. To screen for *Plasmodium* sporozoite-specific rhoptry proteins in the rodent malaria parasite, *Plasmodium berghei*, a proximity-dependent biotin identification method was employed combined with a genome editing strategy. Rhoptry neck protein 12 (RON12) was identified as a rhoptry molecule with the highest transcript levels in sporozoites; and was selected for use as a bait following tagging with UltraID. In RON12::ultraID expressing transgenic sporozoites, several secretory proteins were successfully biotinylated during parasite maturation in mosquitoes, including known rhoptry proteins. A novel rhoptry molecule was identified, PBANKA_1363400, which was localized to sporozoite rhoptries and was predominantly expressed in sporozoites rather than merozoites. This study demonstrates that the UltraID strategy enables highly sensitive and comprehensive protein identification in a species- or stage-specific manner in *Plasmodium* sporozoites.

## 1 Introduction

Malaria parasite infection in humans is initiated by sporozoite inoculation into the skin by anopheline mosquito vectors. Inoculated sporozoites actively migrate through the skin and enter blood vessels to reach the liver, where they cross the sinusoidal cell layer to infect hepatocytes. The molecular mechanisms underlying sporozoite migration to the liver and recognition of target cell hepatocytes have been illuminated through the identification of key secretory proteins, such as TRAP, SPECTs, P36, and P52; which are stored in micronemes, one of the apical organelles (Arredondo et al., 2018; Ishino et al., 2004a, 2004b, 2005; Sultan et al., 1997). Sporozoites infect hepatocytes with the concomitant formation of a surrounding parasitophorous vacuole membrane; within which they develop and proliferate into merozoites, the infective stage of erythrocytes.

The rhoptry is a second apical organelle present in the infective stages of apicomplexan parasites, such as *Plasmodium* merozoites and *Toxoplasma* tachyzoites (Bradley et al., 2005; Sadak et al., 1988; Tokunaga et al., 2019). *Plasmodium* ookinetes are motile but lack rhoptries, and migrate through the epithelial cells of mosquito midguts without establishing intracellular residence nor creating a parasitophorous vacuole - supporting that rhoptry proteins are involved largely in the parasite infection of cells. Specific to *Plasmodium*, the RON complex, composed of RON2, RON4, and RON5, has been demonstrated to be a crucial component in the formation of moving junctions between merozoites and erythrocytes, which enables parasites to enter host cells (Besteiro et al., 2009; Hanssen et al., 2013; Lebrun et al., 2005; Miller et al., 1979; Sam-Yellowe et al., 1988). The mechanism of tight junction formation based on rhoptry proteins is conserved across apicomplexan parasites (Cao et al., 2008; Fernandes et al., 2022; Lamarque et al., 2011; Richard et al., 2010; Srinivasan et al., 2011, 2013). Among 12 examined merozoite rhoptry proteins, 9 were reported to be expressed in oocyst-derived sporozoites (Tokunaga et al., 2019). Conditional gene silencing revealed that RON2, RON4, RON5, and RON11 have important roles during sporozoite invasion of mosquito salivary glands, as well as the infection of hepatocytes (Baba et al., 2023; Bantuchai et al., 2019; Ishino et al., 2019; Nozaki et al., 2020). These rhoptry proteins are suggested to be involved in sporozoite attachment and the onset of motility, both essential for sporozoite migration and target cell invasion (Baba et al., 2023; Ishino et al., 2019).

We hypothesized that some rhoptry proteins might be predominantly expressed in sporozoites, by analogy with RhopH2 and RhopH3 which are dominantly expressed in merozoites. To elucidate stage-specific infection mechanisms, it is necessary to identify stage-specific rhoptry proteins; however, their comprehensive screening has been challenging due to the limited availability of material, as they are obtainable solely by dissection of infected mosquitoes.

Rhoptry proteins were initially identified by organelle purification using *Toxoplasma* tachyzoites and *Plasmodium* merozoites (Perkins, 1992). Recently, the BioID strategy has been developed, and enables the screening of organelle proteins which could not be previously addressed due to limited amounts of parasites, such as osmiophilic bodies in *Plasmodium* sexual stages and dense granules in *Toxoplasma* bradyzoites (Kehrer et al., 2016; Nadipuram et al., 2020). Here, we applied the highly sensitive biotinylating enzyme, UltraID, to screen for novel rhoptry proteins predominantly expressed in sporozoites (Kubitz et al., 2022). RON12 was selected as a bait, as its expression in sporozoites is relatively high among conserved *Plasmodium* rhoptry proteins, and it is dispensable throughout the parasite life cycle (Oda-Yokouchi et al., 2019). Our previous findings indicated that most rhoptry proteins are localized throughout the rhoptries in oocyst-derived sporozoites; and thereby supports the comprehensive screening of rhoptry proteins using sporozoites expressing RON12 fused with UltraID (RON12::ultraID). In this study, we employed mass spectrometry to detect 55 secretory proteins specifically biotinylated by RON12::ultraID, including 13 known rhoptry proteins. Among them, PBANKA_1363400 is dominantly expressed in sporozoites and is localized to rhoptries. By combining the proximity-dependent biotin identification strategy with genome editing, we successfully identified a novel *Plasmodium*-specific and sporozoite-preferred rhoptry protein.

## 2 Materials and methods

### 2.1 Experimental animals, parasites, and mosquitoes

The experimental protocols involving animal subjects were reviewed and approved by the Animal Experiment Committee of the Institute of Science Tokyo. Mice were maintained under controlled environmental conditions with a 12-hour light/dark cycle and ambient temperature. Adult mosquitoes were provided with a 5% (w/v) sucrose solution and maintained at 25°C. All transgenic parasites were derived from the *Plasmodium berghei* ANKA strain and were propagated in female ICR mice, aged 4 to 6 weeks.

### 2.2 RNA isolation, RNA-seq library preparation, and sequencing

Total RNA was extracted from schizont/gametocyte-enriched blood stage parasites, midgut-derived sporozoites, and salivary gland-derived sporozoites. Schizont/gametocyte enrichment was performed by density gradient centrifugation followed by *in vitro* culture of parasite-infected mouse blood. Midgut-derived sporozoites were purified by density gradient centrifugation of a homogenate of oocyst-resident mosquito midguts at day 17 post-feeding using a 17% Accudenz solution (Kennedy et al., 2012) (Accurate Chemical & Scientific Corporation, NY, USA). Salivary glands-derived sporozoites were collected by harvesting the salivary glands of infected mosquitoes at day 21 post-feeding. Total RNA from purified parasites was isolated using TRIzol reagent (Thermo Fisher Scientific, CA, USA) according to the manufacturer’s instructions. RNA-seq libraries were prepared from the total RNA using the NEBNext Ultra II Directional RNA Library Prep Kit for Illumina (New England Biolabs, MA, USA) and sequencing on an Illumina NovaSeq platform (150 bp paired-end reads, approximately 20 million reads per sample). For each stage, three independent biological replicates were performed. Raw sequencing data have been deposited in the DDBJ Sequence Read Archive (DRA) under accession number PRJDB37937.

### 2.3 Analysis of RNA-seq data

The obtained sequence data were mapped to the *P. berghei* ANKA reference genome from PlasmoDB-68 using HISAT2 (version 2.2.1), with the maximum intron length parameter set to 2000. To accurately quantify gene expression, a cDNA-only GFF file was generated from the *P. berghei* ANKA GTF file accessed at PlasmoDB-68. Read counts for each gene were subsequently quantified from the mapped data using featureCounts (version 2.0.1) from the Subread package. Differential gene expression analysis of RNA-seq data of midgut-derived sporozoite against salivary glands-derived sporozoites was performed using the EdgeR R package (version 3.40.2). A generalized linear model (GLM) framework with a negative binomial distribution was employed to account for biological replicates. Differentially expressed genes (DEGs) were defined if their False Discovery Rate (FDR) was less than 0.05 after adjustment using the Benjamini-Hochberg method. Unless otherwise specified, all program parameters were set to their default values. The list of DEGs is described in the **Supplementary Table S1**. The transcripts per million (TPM) values reported for rhoptry molecules in sporozoites are presented in **Supplementary Figure S1**.

### 2.4 Generation of RON12::ultraID or mCherry::ultraID transgenic *Plasmodium berghei*

To generate transgenic parasites expressing RON12 tagged with AGIA-ultraID at its C-terminus, the endogenous locus of the targeted RON12 gene in the GFP-Akaluc (GFPAka) parental line, constitutively expressing GFP and Akaluc separated by a T2A skip peptide, was transfected with linearized donor DNA for homology directed repair and Cas9 nuclease and single guide RNA (sgRNA) expressing plasmid using CRISPR/Cas9 system, as described with some modifications (Shinzawa et al., 2020). The donor DNA was generated by overlap extension PCR to fuse the left and right homology arms and ultraID-coding DNA. The left and right homology arms were amplified from genomic DNA using RON12-LH-F/RON12-LH-R and RON12-RH-F/RON12-RH-R, respectively. The ultraID-coding DNA was synthesized by a gene synthesis service (GeneArt, Thermo Fisher Scientific) with long linker (GGGGSGGGGSGGGGSPDPPVA) and an AGIA epitope tag (EEAAGIARPL), and amplified with ultraID-F and ultraID-R. These three PCR fragments were fused by overlap extension PCR, and the fused DNA fragments were used as linear donor DNA for genome editing. The psgRNA-Cas9 plasmid was used for Cas9 and sgRNA expression. This plasmid contains both the Cas9 and sgRNA expression cassettes and was generated by removing the centromere sequence from the pCas9-U6-hycen plasmid. Candidate 20 bp guide RNA sequences were selected using the CHOPCHOP online tool (https://chopchop.cbu.uib.no), based on predicted high on-target efficiency and minimal off-target potential. A pair of complementary oligonucleotides (PbRON12-gRNA-F and PbRON12-gRNA-R) corresponding to the selected guide RNA sequence were annealed and ligated into the BsmBI-digested psgRNA-Cas9 plasmid. The resulting plasmids were used for genome editing as Cas9/sgRNA plasmids.

As a control line, an mCherry::ultraID constitutive expression cassette was inserted into the dispensable *p230p* locus. The donor DNA was generated as plasmid DNA which contains left and right *p230p* homology arms and a *pbef1α* promoter driving an ultraID-fused mCherry expression cassette. The left and right homology arms were amplified from genomic DNA with p230p-LH-F/p230p-LH-R and p230p-RH-F/p230p-RH-R, respectively, and inserted into KpnI/NotI-digested pBlueScript II SK by In-Fusion cloning (Takara Bio USA, CA, USA) to generate pBSSK-p230pHR. The ef1α promoter and the dhfr-ts 3’UTR were amplified from the genomic DNA with ef1α-F/ef1α-R and 3’dhfr-F/3’dhfr-R. The mCherry2 coding DNA was amplified from mCherry2-C1 (Addgene plasmid #54563) with mCh-F and mCh-R. The ultraID-coding DNA was amplified from the synthesized DNA as described above with ultraID-F2 and ultraID-R2. These four DNA fragments were inserted into PmlI/SalI-digested pBSSK-p230pHR to generate pBSSK-p230pHR-ef-mCh-ultraID. The resulting plasmid was linearized by digestion with KpnI/NotI and was used as donor DNA for genome editing. p230p-sgRNA_d3-F and p230p-sgRNA_d3-R (Shinzawa et al., 2020) were cloned into psgRNA-Cas9 to generate the Cas9/sgRNA plasmid.

Parasite transfections were performed as described with some modifications (Janse et al., 2006; Shinzawa et al., 2020). Roughly 2 × 10^7^ purified GFPAka parental schizonts were electroporated with 5 μg of linearized donor DNA and Cas9/sgRNA plasmid DNA using a Lonza 4D-Nucleofector Core Unit with the FI-115 program. Transfected parasites were immediately injected intravenously into a naïve ICR mouse and then selected with pyrimethamine (70 μg/mL, MP biomedicals, Illkirch, France) in drinking water, at 30 h post-infection for 5 days, followed by withdrawal of drug. After confirming the emergence of parasites in the peripheral blood, DNA integration occurring at the target locus by homology-directed repair was confirmed by genotyping PCR. Clonal transgenic parasites were obtained by limiting dilution procedures. Oligonucleotides used for the plasmid construction and genotyping PCR were described in **Supplementary Table S3**.

### 2.5 Western blotting

Protein homogenates were derived from oocyst-derived sporozoites harvested at 14 days post-feeding or schizont-enriched parasites by density gradient centrifugation after *in vitro* culture of infected mouse blood (Janse et al., 2006). The homogenates were solubilized in SDS-PAGE loading buffer and electrophoretic separation was performed using a specified quantity of parasites on 5–20% polyacrylamide gradient gels (ATTO, Tokyo, Japan), followed by transfer to polyvinylidene difluoride (PVDF) membranes using a semi-dry system. Membranes were blocked overnight with 4% skim milk and subsequently incubated with primary antibodies at the following dilutions: rabbit anti-AGIA antibodies (1:1,000 or 1:4,000 dilution; Morita et al., 2021; Yano et al., 2016), mouse anti-RON12 antibodies (1:4000 dilution; Oda-Yokouchi et al., 2019), and rabbit anti-HSP70 antibodies (Ishino et al., 2019) for 90 minutes at room temperature. Horseradish peroxidase (HRP)-conjugated goat anti-rabbit IgG (H+L) (11-035-144, 1:10,000 dilution, Jackson ImmunoResearch Laboratories, PA, USA) and HRP-conjugated goat anti-mouse IgG (H+L) (115-035-146, 1: 10,000 dilution, Jackson ImmunoResearch laboratories) were applied as secondary antibodies for 60 minutes under identical conditions. Chemiluminescent detection was conducted using Immobilon Western Chemiluminescent HRP Substrate (Millipore, MA, USA), with signals visualized and captured via the iBright CL1500 Imaging System (Thermo Fisher Scientific).

### 2.6 Indirect immunofluorescence analysis

Oocyst-derived sporozoites were dissected at day 14 post-feeding, and 1000 parasites were seeded on a ten-well slide, dried with a dryer, fixed with 4% PFA for 20 minutes, and kept in Dulbecco’s PBS (D-PBS) at 4°C until use. IFA was conducted by initially blocking the slides with Blocking One Histo (Nacalai Tesque, Kyoto, Japan) for 15 to 30 minutes at room temperature. Primary antibodies were applied at the following dilutions: chimeric rabbit-mouse anti-AGIA antibodies (1:1,000 dilution), rabbit anti-AGIA antibodies (1:1000 dilution), rabbit anti-RON4 antibodies (1:20,000 dilution; Nozaki et al., 2020), and mouse anti-biotin antibodies (1:500 dilution; Rockland Immunochemicals, PA, USA), with incubation overnight at 4°C. For visualization, secondary antibodies conjugated with fluorophores were employed. Goat anti-Mouse IgG (H+L) Cross-Adsorbed Secondary Antibody, Alexa Fluor plus 555 (1:500 dilution; Thermo Fisher Scientific) and Goat anti-Rabbit IgG (H+L) Cross-Adsorbed Secondary Antibody, Alexa Fluor plus 647 (1:500 dilution; Thermo Fisher Scientific) were used as secondary antibodies. Nuclei were labelled using Hoechst 33342 (0.5 µg/ml; Thermo Fisher Scientific). Image acquisition was performed using Stralis 5 confocal microscopy (Leica, Wetzlar, Germany), and subsequent pseudo-coloring was carried out using ImageJ software.

### 2.7 Biotin labeling and sporozoite collection

Female ICR mice infected with either RON12::ultraID or mCherry::ultraID were anesthetized and used to feed overnight-starved female *Anopheles stephensi* mosquitoes for 60 to 120 minutes at 20 °C. Parasite-infected mosquitoes were maintained on a 5% sucrose diet until day 11 post-feeding, and subsequently with 10 mM biotin (Nacalai Tesque, Kyoto, Japan) dissolved in 5% sucrose to facilitate biotinylated protein capture. On day 14 post-feeding, oocyst-derived sporozoites were obtained by dissection. For biotinylated protein detection, sporozoite-containing midgut homogenates were dissolved in an SDS-PAGE loading buffer with 5% β‐mercaptoethanol. Electrophoresis was performed using a specified quantity of parasites on 5–20% polyacrylamide gradient gels, followed by transfer to PVDF membranes using a semi-dry system. Membranes were blocked overnight with 4% skim milk and incubated with streptavidin conjugated to horseradish peroxidase (1:2,000 dilution; Proteintech, IL, USA) for 90 minutes at room temperature. Chemiluminescent detection was conducted using Immobilon Western Chemiluminescent HRP Substrate (Millipore), with signals visualized and captured via an iBright CL1500 Imaging System (Thermo Fisher Scientific).

### 2.8 Mass spectrometry

The ultraID-based proximity-dependent biotin identification technique was performed according to a published protocol (Nishino et al., 2022). The oocyst-derived sporozoites of either RON12::ultraID or mCherry::ultraID from biotin-fed infected mosquitoes were obtained by dissection as described above. Three independent samples, each containing more than three million sporozoites, were lysed with guanidine-TCEP buffer (6 M guanidine-HCl, 100 mM HEPES-NaOH, pH 7.5, 10 mM TCEP, 40 mM chloroacetamide). Proteins in the lysates were digested with trypsin, and biotinylated peptides were enriched using Tamavidin 2-REV magnetic beads (FUJIFILM Wako Pure Chemical Corporation, Osaka, Japan), followed by desalting with GL-Tip SDB (GL Sciences, Eindhoven, Netherlands). The resulting peptides were analyzed by nanoLC-MS/MS on an EASY-nLC 1200 system coupled to an Orbitrap Fusion mass spectrometer (Thermo Fisher Scientific). The raw data were searched with Proteome Discoverer (ver. 2.5) against the *P. berghei* ANKA strain protein database (PlasmoDB) using the Sequest HT search engine. Label-free quantification was performed with the precursor ion quantifier node with normalization across samples.

### 2.9 Immuno-electron microscopy

Cultivated schizonts were purified by density gradient centrifugation from transgenic parasites expressing AGIA-tagged rhoptry candidate proteins. Mosquito midguts infected with transgenic parasites expressing AGIA-tagged rhoptry candidate proteins were collected by dissection at day 16 post-feeding. The samples were subsequently fixed with 1% paraformaldehyde and 0.2% glutaraldehyde in 0.1 M Hepes buffer and then dehydrated and embedded in LR white resin. Ultrathin sections were blocked for 30 min in 0.1M PBS containing 5% nonfat dry milk and 0.01% Tween 20 (PBSmilk‐Tween), followed by overnight incubation with rabbit anti-AGIA antibodies (1:100 dilution; Morita et al., 2021) in PBS milk‐Tween. After washing with PBS containing 0.4% Block Ace and 0.01% Tween 20 (PBS Block Ace-Tween), samples were incubated for 90 min in PBS milk‐Tween containing goat anti‐rabbit IgG conjugated to 15 nm of gold particles. The grids were then rinsed with PBS Block Ace-Tween followed by distilled water. The grids were dried and stained with 2% uranyl acetate in 50% methanol and lead citrate. Samples were examined with a transmission electron microscope (JEM-1230; JEOL, Tokyo, Japan).

### 2.10 Generation of AGIA-tagged proteins expressing parasites

To generate transgenic parasites expressing C-terminal AGIA-tagged proteins, the native locus of the targeted molecule in the GFPAka genome was replaced by single crossover homologous recombination. Schematic representation of the transgenic vector construction is shown in **Supplementary Figure S4**. Two partial Sel1-encoding DNA fragments were amplified by Sel1-F1/Sel1-R1 and Sel1-F2/Sel1-R2. The Sel-R2 included AGIA-tag-encoding sequences. The Sel1-F2/Sel1-R2 fragment was then used for subsequent amplification with Sel1-F2/Sel1-R3. The Sel1-F1/Sel1-R1 fragment and the Sel1-F2/Sel1-R3 fragment were inserted by In-Fusion cloning into the XhoI/BglII site of pSK-1, which is the GFP-tagging vector including a hDHFR expression cassette (Yuda et al., 2009). The resultant plasmid was named pSC-Sel1::AGIA. Subsequently, pairs of partial DNA fragments encoding CA, CRMP4, and Pb13634 were amplified by specific primers, and inserted into the XhoI/NheI site of pSC-Sel1::AGIA. The resulting plasmids were named pSC-CA::AGIA, pSC-CRMP4::AGIA, and pSC-Pb13634::AGIA, respectively. The recombinant plasmids were linearized by SmaI to cleave DNA sequences encoding candidate genes. Electroporation of 10 μg linearized DNA into schizont-enriched GFPAka and selection of transgenic parasites were performed as described above. Thereafter, continuous drug selection was initiated at 30 h post-inoculation to maintain the plasmid integration of transgenic parasites. DNA integration into the target locus was confirmed by PCR genotyping, and transgenic parasites were isolated by limiting dilution. Oligonucleotides used for the plasmid construction and genotyping PCR are described in **Supplementary Table S3**.

### 2.11 DiCre-mediated target gene excision of PBANKA_1363400

To achieve conditional target gene excision, the dimerisable Cre recombinase (DiCre) system was employed by the generation of DiCre-constitutive expressing transgenic lines with the integration of the DiCre expression cassette into dispensable intergenic regions within chromosome 5 (Sekine et al., submitted). The DNA sequence encoding the DiCre expression cassette was amplified with DiCre-F and DiCre-R from the DiCre plasmid as described (Fernandes et al., 2020), and inserted into SpeI/SalI sites of the plasmid containing the homology-arms of the dispensable intergenic regions. DiCre-cassette plasmids linearized using KpnI and NotI and the Cas9/sgRNA plasmid targeting the dispensable intergenic locus were used for the transfection of the GFPAka parental parasite line using the genome editing procedures described above. Correct integration of the DiCre cassette was confirmed by PCR amplification using locus-specific primers, with amplicon size analysis confirming precise cassette insertion. After establishment of parasite clones by limiting dilution, the resultant parasite line was named GFPAka-DiCre, which was used as a parental line for subsequent genome editing as follows.

In the initial subsequent step, two loxP sites were introduced into the PBANKA_1363400 (*Plasmodium specific rhoptry protein 1, prp1*) locus: one within the 3’ UTR and the other within the coding region, embedded in an artificial intron along with an AGIA epitope tag at the C-terminus of PRP1. To construct the donor DNA plasmid, a DNA fragment consisting of partial sequences of the *PRP1* C-terminus, the AGIA tag, and the loxP sequence (ATAACTTCGTATAGCATACATTATACGAAGTTAT) was synthesized (GeneArt, Thermo Fisher Scientific), then amplified with prp1-HR2-F/prp1-HR2-R and cloned into the HindIII/EcoRI sites of pBlueScript II SK (+) (Stratagene, CA, USA). Following this, the 3’-UTR of *prp1* was inserted into the EcoRI/BamHI sites of the resultant plasmid, which was named pBSSK-AGIA-loxP-prp1HR2. Subsequently, three fragments representing the 5’ UTR and the first 5 bp of the *prp1* coding sequence, a synthesized artificial intron from the *slarp* gene containing a loxP site (GeneArt, Thermo Fisher Scientific) (Das et al., 2025), and the rest of the *prp1* genomic sequence were inserted into the HindIII/NheI sites of pBSSK-AGIA-loxP-prp1HR2; resulting in the final construct, pBSSK-prp1-2x[loxP]_donor. For the above three amplifications, the following six primers were used: prp1-HR1-F, prp1-HR1-R, loxPint[slarp]-F, loxPint[slarp]-R, prp1-F, and Pb13634-R2.

The plasmid expressing Cas9 and two sgRNAs, psgRNA2-Cas9, was used for the introduction of loxP into the *prp1* locus. The psgRNA2-Cas9 plasmid, which contains an additional sgRNA expression cassette, was constructed by inserting a *Plasmodium yoelii* U6 promoter (PY17X_1359900) and an sgRNA scaffold sequence amplified with PyU6-F/PyU6-R and sgRNA-scaffold-F/sgRNA-scaffold-R into the SacII site of the psgRNA-Cas9 plasmid, following the introduction of a synonymous mutation at the BsaI site in β-lactamase. Complementary oligonucleotides corresponding to the guide RNA were annealed and ligated into the digested psgRNA-Cas9 with BsmBI or BsaI. The resulting plasmids were used for genome editing as Cas9/sgRNA plasmids.

Linearized pBSSK-prp1-2x[loxP]_donor digested with SalI/NotI and Cas9/sgRNA plasmids were used for the transfection of the GFPAka-DiCre line. Correct integrations of the loxP sites were confirmed by genotyping PCR using locus-specific primers and subsequent Sanger sequencing. Transgenic parasite clones PRP1-2x[loxP] were obtained by the limiting dilution method. Oligonucleotides used for the plasmid construction and genotyping PCR are described in **Supplementary Table S3**.

Excision at the loxP sites was induced by the addition of 10 nM rapamycin during *in vitro* culture of erythrocytic-stage PRP1-2x[loxP] parasites, and excision events were assessed by PCR and Sanger sequencing at 16 hours post-treatment. The excised parasite (ΔPRP1) was intravenously inoculated into female ICR mice, and when the parasitemia reached 1-3%, blood was collected by cardiac puncture and preserved at -80 °C for further functional analysis.

### 2.12 *In vivo* blood stage parasite proliferation assay

Cryopreserved *P. berghei* infected erythrocytes with either GFPAka-DiCre or ΔPRP1 parasites were intraperitoneally injected into female ICR mice to obtain asexual stage parasites. When the parasitemia within tail blood reached 0.2-0.5%, then 20,000 infected erythrocytes were administered via intravenous injection into female ICR mice (n = 5 per group). Parasitemias were quantitatively evaluated beginning at 72 hours post-infection through daily microscopic analysis of Giemsa-stained thin smears derived from mouse tail blood.

### 2.13 Sporozoite invasion and infection assays

To assess the impact of the ΔPRP1 parasite genotype on oocyst development and sporozoite invasion into salivary glands, female *Anopheles stephensi* mosquitoes were fed on infected female ICR mice with either GFPAka-DiCre or ΔPRP1 parasites. Mosquitoes exhibiting full engorgement were isolated and maintained at 20 °C until dissection. Mosquito containers demonstrating >80% oocyst prevalence, as quantified by microscopic examination of midguts between days 8–12 post-feeding, were selected for further analysis. At days 20 to 23 post-infection, sporozoites were harvested from either midguts or salivary glands via dissection followed by mechanical homogenization of the respective tissues to release sporozoites. Subsequently, sporozoite numbers per mosquito were determined. The experiments were repeated more than three times. To assess the effect of the disruption of PRP1 on hepatocyte infection, 15,000 salivary gland sporozoites from each parasite were intravenously inoculated into 5-weeks old female C57BL/6 mice (n=4 per group). The parasitemia progression was assessed beginning on day 3 post-inoculation using daily Giemsa-stained thin smears obtained from mouse tail blood.

### 2.14 Chimeric rabbit-mouse anti-AGIA antibody preparation

The variable regions of the heavy and light chains of rabbit anti-AGIA antibodies (Yano et al., 2016) were fused with the constant regions of mouse IgG1 and light chain cDNA, respectively, and subcloned into the pcDNA3.4 expression vector. Then, the chimeric rabbit-mouse anti-AGIA antibody was expressed using the Expi293F Expression System (Thermo Fisher Scientific) according to the manufacturer’s instructions. The secreted antibody was purified from the culture medium using protein G Sepharose 4 Fast Flow (Cytiva, MA, USA), and the buffer was exchanged using a PD-10 column (Cytiva).

### 2.15 Statistics

Statistical analyses were conducted using GraphPad Prism version 9.0.0 (GraphPad Software).

## 3 Results

### 3.1 Preparation of a transcriptome database for schizonts/gametocytes, oocyst-derived sporozoites, and sporozoite-invaded salivary glands

To support screening for novel rhoptry proteins in sporozoites, firstly a transcriptome data set was prepared by RNA seq and containing oocyst-derived sporozoites and sporozoite-invaded salivary glands, both collected from *P. berghei* ANKA infected *A. stephensi*. For comparison, an RNA seq dataset was generated using schizonts/gametocytes-enriched *P. berghei* parasites. As shown in **Supplementary Table S1**, all known sporozoite rhoptry molecules, except for RON5, have high transcripts per million (TPM) in oocyst-derived sporozoites compared to those in salivary gland sporozoites. This is consistent with our previous report showing that the mRNA amounts of most rhoptry molecules increase during sporozoite formation in oocysts, then decrease after salivary gland invasion (Tokunaga et al., 2019). *ron12* transcript levels are the highest in sporozoites among known *Plasmodium* rhoptry molecules (**Supplementary Figure S1**). RON12 was selected as a likely abundant bait to screen for novel rhoptry proteins in sporozoites by BioID, because it is a non-essential protein and is thereby predicted to be amenable to genetic manipulation, and additionally, specific antibodies are available which recognize it (Oda-Yokouchi et al., 2019).

### 3.2 Generation of ultraID tagging PbRON12 expressing transgenic parasites to screen for novel rhoptry proteins by proximity-dependent biotin identification

**Figure 1:**
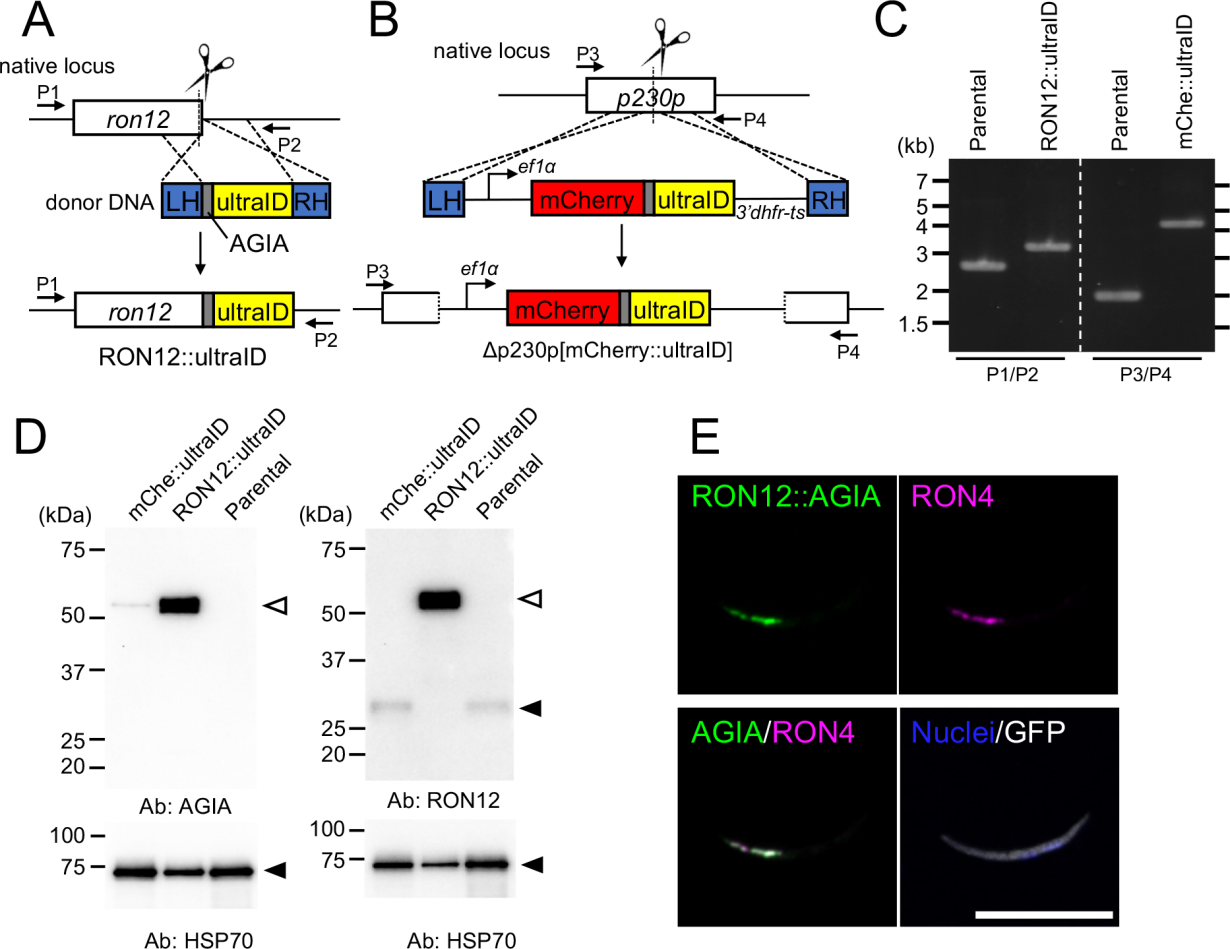
Generation of *Plasmodium berghei* transgenic parasites which express RON12::ultraID and mCherry::ultraID. **(A)** Schematic representation of the generation of transgenic *Plasmodium berghei* parasite lines expressing RON12::ultraID at the native *ron12* locus using the CRISPR/Cas9 system. The donor DNA used for transfection is shown in the middle. Scissors indicate the sites of CRISPR/Cas9 recognition. After homology directed repair, the coding sequences of AGIA and ultraID were integrated at the end of the RON12 coding region (bottom). The transgenic parasites were designated RON12::ultraID. **(B)** Generation of a control transgenic parasite line expressing cytosolic mCherry fused to ultraID with an AGIA tag. The donor DNA contains an expression cassette encoding mCherry::AGIA::ultraID, driven by the *Plasmodium berghei* elongation factor 1 alpha promoter (*ef1α*), followed by the 3’UTR of *dhfr-ts*. This expression cassette was integrated into the dispensable *p230p* locus by CRISPR/Cas9 dependent homology directed repair. The resulting control parasite line was designated Δ230p[mCherry::ultraID] (mChe::ultraID). **(C)** DNA integrations into the expected sites were investigated by PCR genotyping using cloned transgenic parasite genomic DNA as templates. Primers for detection are indicated in **(A)** and **(B)**. The primer set P1/P2 amplified DNA fragments at approximately 3 kbp and 3.5 kbp, from the parental line and RON12::ultraID genomic DNA, respectively. The increase in DNA fragment size corresponds to the correct integration of the AGIA::ultraID sequence at the *ron12* locus, changing from 2,693 bp to 3,353 bp. Similarly, primer set P3/P4 amplified a DNA fragment from mChe::ultraID transgenic parasites approximately 2 kbp longer compared to that of the parental line, thus confirming the successful integration of the mCherry::AGIA::ultraID expression cassette. **(D)** UltraID-tagged proteins were expressed in each transgenic parasite line. Western blotting of 30,000 sporozoites collected from infected mosquito midguts were performed using rabbit anti-AGIA antibodies (1:4,000 dilution, left panel) or using anti-RON12 antibodies (1:4,000 dilution, right panel) to detect ultraID-fused proteins or RON12 in transgenic parasite lines and the parental line. (Left) Open arrowhead indicates specific bands at approximately 50 kDa, corresponding to RON12::ultraID and mChe::ultraID. (Right) Open and closed arrowheads indicate the RON12::ultraID in RON12::ultraID lines and native RON12 in mChe::ultraID and parental lines. Parasite protein loading was confirmed using anti-HSP70 antibodies (1:100,000 dilution, lower panels). The sizes of the molecular weight markers are shown on the left. **(E)** Indirect-immunofluorescent analysis of RON12::ultraID in oocyst-derived sporozoites. Midgut sporozoites were collected at day 14 post-feeding, fixed with 4% PFA for 20 minutes, followed by incubation with 0.1% Triton X-100. Samples were then incubated with chimeric rabbit-mouse anti-AGIA antibodies (1:1,000 dilution, shown in green) and rabbit anti-RON4 antibodies (1:20,000 dilution) as a rhoptry marker (magenta). Cytoplasmic GFP expression is shown in white and nuclei were stained with Hoechst (0.5 µg/ml, blue). Scale bar = 10 μm.

For the identification of novel rhoptry proteins in sporozoites, ultraID was selected as a proximity-dependent biotin enzyme due to its advantageous features such as high reactivity and small size (Kubitz et al., 2022). To label rhoptry proteins during sporogony in oocysts *in vivo*, a transgenic *P. berghei* parasite line was generated to express a C-terminally ultraID-fused RON12 protein by knock-in at the endogenous *ron12* locus using the CRISPR/Cas9 system (Jinek et al., 2012; Lee et al., 2019; Shinzawa et al., 2020). To monitor protein expression, the epitope tag AGIA was inserted between RON12 and ultraID (**Figure 1A**) (Morita et al., 2021; Yano et al., 2016). The resulting transgenic parasite line was named RON12::ultraID. As a control line, an expression cassette of mCherry tagged by AGIA and ultraID under control of an *ef1a* promoter was inserted into the dispensable gene, *p230p*, in the same parental parasite line (**Figure 1B**, Δp230p[mCherry::ultraID]; hereafter mChe::ultraID). PCR genotyping using genomic DNA of cloned transgenic parasites, RON12::ultraID and mChe::ultraID, demonstrated that successful DNA integration at the expected sites occurred in both transgenic parasite lines (**Figure 1C**). Expression of RON12::ultraID and mChe::ultraID in transgenic sporozoites was examined by western blotting. Sporozoites were collected by harvesting mosquito midguts at day 14 post-feeding of transgenic or parental parasite-infected mice. Anti-AGIA antibodies detected RON12::ultraID and mChe::ultraID proteins as major bands at approximately 50 kDa in each transgenic sporozoite line, corresponding to their calculated molecular weights of 49.4 kDa and 49.9 kDa, respectively (**Figure 1D**, left panels). UltraID fusion to RON12 was confirmed using anti-RON12 antibodies, showing that its native 27 kDa band detected in mChe::ultraID sporozoites was shifted to a band of about 50 kDa in RON12::ultraID sporozoites (**Figure 1D**, right panels). RON12::ultraID localization in oocyst-derived sporozoites was investigated by immunofluorescence analysis (IFA) using anti-AGIA antibodies, together with anti-RON4 antibodies as a rhoptry marker (Nozaki et al., 2020). RON12::ultraID was detected at the sporozoite apical end and colocalized with RON4, indicating that RON12::ultraID was correctly transported to rhoptries (**Figure 1E**, Oda-Yokouchi et al., 2019). RON12::ultraID parasites proliferated as efficiently as control parasites in mouse blood and formed a similar number of sporozoites within mosquito midguts (**Supplementary Figure S2**); thus demonstrating that UltraID tagging of RON12 did not affect the parasite lifecycle. This RON12::ultraID transgenic line was then used to screen novel sporozoite rhoptry proteins by proximity-dependent biotin identification in *P. berghei.*

### 3.3 Detection of biotinylated proteins by RON12::ultraID in oocyst-derived sporozoites

**Figure 2:**
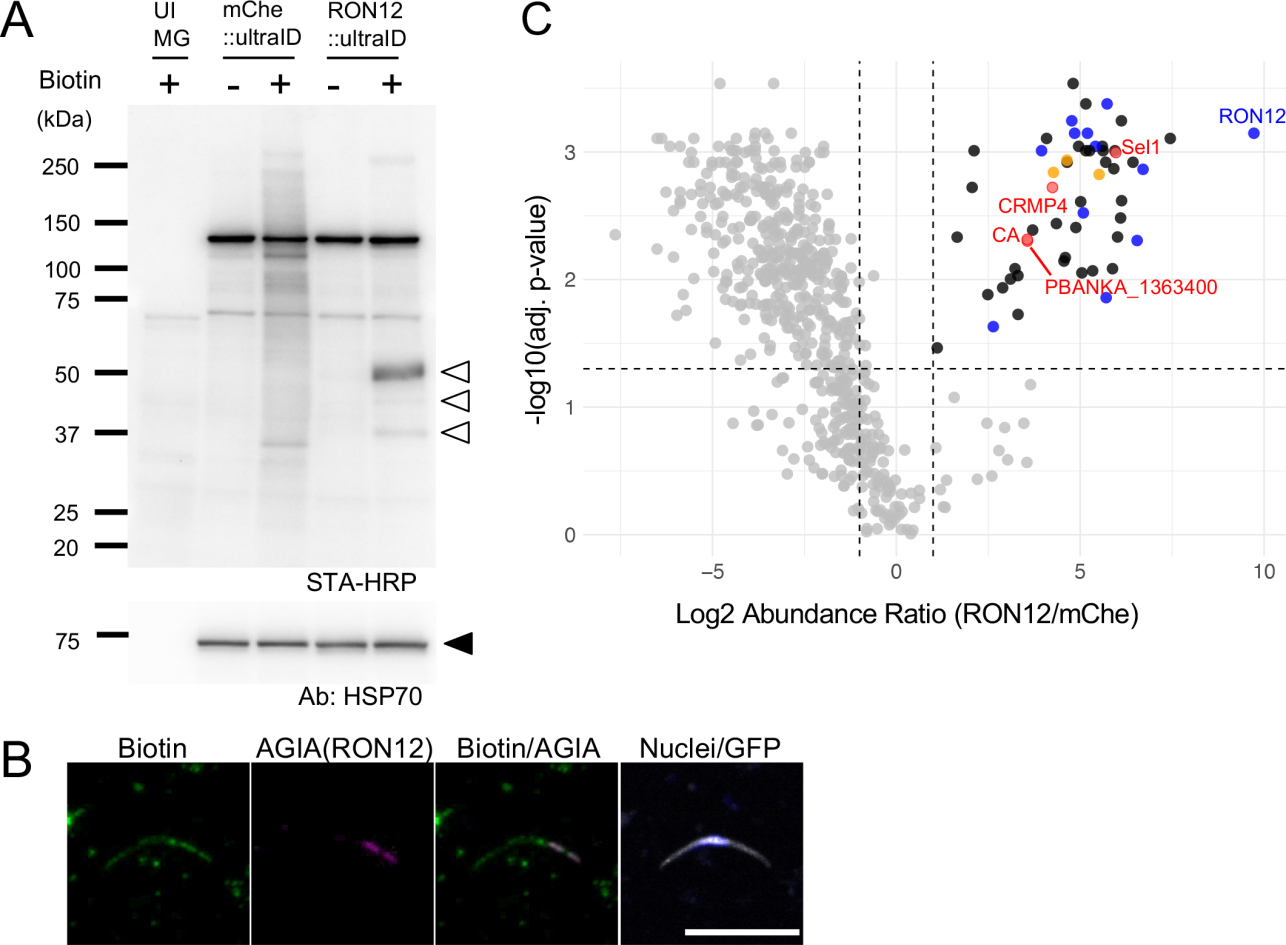
Detection of specifically biotinylated proteins by RON12::ultraID in sporozoites. **(A)** Mosquitoes infected with RON12::ultraID or mChe::ultraID parasites were supplied with (+) or without (-) 10 mM biotin containing 5% sucrose solution for three days from day 11 post-feeding. Sporozoites were collected by harvesting infected mosquito midguts and protein lysates of 100,000 sporozoites were subjected to SDS-PAGE, followed by transfer to a PVDF membrane. To assess non-specific biotinylation in mosquito midgut tissues, four midguts of uninfected mosquitoes, fed with 10 mM biotin containing 5% sucrose solution for 3 days, were loaded as above (shown as UI MG). Biotinylated proteins were detected by incubating with streptavidin conjugated to horseradish peroxidase (1:2,000 dilution). Open arrowheads indicate the specifically biotinylated proteins by RON12::ultraID, including self-biotinylated protein at approximately 50 kDa. The same PVDF membrane was incubated with anti-HSP70 antibodies (1:1,000,000 dilution) to demonstrate parasite protein loading. The expected HSP70 protein is indicated by a closed arrowhead. The sizes of the molecular weight markers are shown on the left. **(B)** IFA detection of biotinylated proteins by RON12::ultraID in oocyst-derived sporozoites. RON12::ultraID sporozoites were collected from mosquito midguts at day 14 post-feeding after three days of biotin treatment, and fixed with 4% PFA, followed by 0.1% Triton X-100 treatment. Sporozoites were incubated with mouse anti-biotin antibodies (1:500 dilution, shown in green) together with rabbit anti-AGIA antibodies (1:1,000 dilution, magenta) to show RON12::ultraID localization. GFP expressed in cytosol (white) and nuclei stained with Hoechst (0.5 µg/ml, blue) are shown. Scale bar = 10 μm. **(C)** Volcano plot demonstrating specifically biotinylated proteins by RON12::ultraID (shown in black). The X-axis depicts the ratio of detected peptides between RON12::ultraID and mChe::ultraID. The P-values from three independent samples are shown on the Y-axis. Known rhoptry proteins which were RON12::ultraID-dependently biotinylated are indicated in blue. Selected and non-selected candidate proteins are shown in red and yellow, respectively.

To investigate proteins specifically biotinylated by ultraID fused to RON12 in oocyst-derived sporozoites, female *A. stephensi* mosquitoes were fed on mice infected with either RON12::ultraID or mChe::ultraID parasites. From days 11 to 14 the infected mosquitoes were fed sucrose with 10 mM biotin, at which point midguts were harvested to detect *in vivo* biotinylated sporozoite proteins. It was confirmed that administration of 10 mM biotin for three days had no impact on oocyst development and sporozoite formation inside oocysts (**Supplementary Figure S3**). Biotinylated proteins in RON12::ultraID or mChe::ultraID sporozoites were detected by electrophoretic separation and incubation of western transfer PVDF membranes with HRP-conjugated streptavidin (**Figure 2A**). Upon biotin administration, several specific signals were detected in both transgenic sporozoites lysates, indicating that biotin was efficiently delivered into sporozoites. The biotinylated proteins detected in midguts collected from uninfected mosquitoes fed with biotin were natively biotinylated mosquito proteins. By comparison to mChe::ultraID and uninfected mosquito midgut samples, several proteins were biotinylated uniquely by RON12::ultraID, including the self-biotinylated RON12::ultraID. The accumulation of biotinylated proteins at the apical end in RON12::ultraID oocyst-derived sporozoites was detected by IFA using anti-biotin antibodies (**Figure 2B**). In summary, three days of biotin delivery via the sucrose water for parasite-infected mosquitoes appeared to provoke the ultraID enzyme fused to RON12 to efficiently labeled proteins in proximity during sporozoite maturation in oocysts, and likely within rhoptries of *Plasmodium* sporozoites. Therefore, oocyst-derived sporozoites were collected for both RON12::ultraID and mChe::ultraID for mass spectrometry analysis to detect sporozoite proteins specifically biotinylated by RON12::ultraID.

### 3.4 Screening for proteins specifically biotinylated by RON12::ultraID using mass spectrometry analysis

Biotinylated peptides were enriched using Tamavidin2-Rev beads from triplicate samples of RON12::ultraID and mCh::ultraID sporozoites and then analyzed by LC-MS/MS (Kim et al., 2016). Fifty-five *Plasmodium* proteins were significantly biotinylated by RON12::ultraID at levels at least two-fold higher compared to mChe::ultraID; including 34 putative secretory proteins possessing an N-terminal signal peptide (**Figure 2C**, **Supplementary Table S2**). Thirteen known rhoptry proteins were found in the selected gene list; specifically, rhoptry-associated membrane antigen (RAMA), rhoptry neck proteins 2 (RON2), RON3, RON4, RON5, RON6, RON12, rhoptry-associated leucine zipper-like protein 1 (RALP1), rhoptry-associated protein 2/3 (RAP2/3), rhoptry protein 14 (ROP14), rhoptry-associated protein 1 (RAP1), apical asparagine-rich protein (AARP), and dipeptidyl aminopeptidase 3 (DPAP3) (**Figure 2C**, shown in blue dots, **Supplementary Table S2**) (Baldi et al., 2000, 2002; Bradley et al., 2005; Crewther et al., 1990; Ghosh et al., 2019; Haase et al., 2008; Ishino et al., 2019; Oda-Yokouchi et al., 2019; Proellocks et al., 2009; Risco-Castillo et al., 2014; Tokunaga et al., 2019; Topolska et al., 2003; Wickramarachchi et al., 2008; Zhao et al., 2014). These results strongly indicated that the proximity-dependent biotinylation approach using RON12::ultraID successfully captured rhoptry proteins in sporozoites *in vivo*.

Taken together with information obtained from RNA-seq indicating that the transcription levels of most rhoptry proteins are higher in oocyst-derived sporozoites (**Supplementary Table S1**), the following criteria were set for the identification of candidate novel rhoptry proteins predominantly expressed in sporozoites: 1) significantly and at least two times more biotinylated in RON12::ultraID sporozoites than in mChe::ultraID sporozoites by LC-MS/MS; 2) conserved among *Plasmodium* spp with orthologues in *Plasmodium falciparum* and *Plasmodium vivax*; 3) containing an N-terminal signal peptide predictive of secretion; 4) transcript levels in sporozoites similar to known rhoptry molecules, such as greater than *ron4* (25 TPM in midgut sporozoites); and 5) transcript levels higher in midgut sporozoites than in salivary gland sporozoites. Proteins were excluded from the candidate list whose localizations were previously demonstrated in rhoptries or other organelles.

According to these criteria, seven proteins were selected for further investigation (**Table 1**). Examples of excluded proteins whose localization have been reported include parasite-infected erythrocyte surface protein, secreted ookinete protein, and glideosome-associated protein 50 (Parkyn Schneider et al., 2017; Saini et al., 2017). As a result, four proteins were selected as candidates for novel rhoptry proteins expressed in sporozoites; namely, PBANKA_0301900 (Sel1 repeat-containing protein, Sel1), PBANKA_0909000 (carbonic anhydrase, CA), PBANKA_1300800 (cysteine repeat modular protein 4, CRMP4), and PBANKA_1363400 (Pb13634).

**Table 1:** List of specifically detected biotinylated proteins in RON12::ultraID expressing sporozoites.

Gene ID	Description	Name	Abundance Ratio^#^	Adj. P-Value^#^	MW (kDa)	SchGam^†^	mgSPZ^†^	sgSPZ^†^	References
PBANKA_0209200	parasite-infected erythrocyte surface protein	PIESP15	24.91	1.2E-03	58.8	17.41	26.19	4.63	Florens et al., 2004
PBANKA_0301900	Sel1 repeat-containing protein, putative	Sel1	62.51	1.0E-03	214.1	42.32	476.41	51.23	This study
PBANKA_0619200	secreted ookinete protein, putative	PSOP1	45.73	1.5E-03	54.7	478.47	102.21	2.40	Tachibana et al., 2021; Ecker et al., 2008
PBANKA_0819000	glideosome-associated protein 50, putative	GAP50	19.33	1.4E-03	44.3	1222.92	65.99	2.87	Baum et al., 2006
PBANKA_0909000	carbonic anhydrase, putative	CA	11.82	5.0E-03	75.2	6.73	74.57	1.34	This study
PBANKA_1300800	cysteine repeat modular protein 4	CRMP4	18.98	1.9E-03	632.5	11.60	305.86	24.40	Thompson et al., 2007, This study
PBANKA_1363400	conserved Plasmodium protein, unknown function		11.80	4.8E-03	29.6	86.72	80.02	1.36	This study

#Mass spectrometry of biotinylated peptides.

†TPM calculated by RNA-sequencing.

Gene ID obtained from the PlasmoDB database (https://plasmodb.org/plasmo/app).

### 3.5 Characterization of four candidate proteins in sporozoites and schizonts

To investigate the expression and subcellular localization of the four candidate proteins in *P. berghei*, a single-crossover homologous recombination strategy was used to generate transgenic parasite lines which expressed each protein with an AGIA epitope tag at its C-terminus (**Supplementary Figure S4A**). Successful DNA integration to create chimeric proteins was confirmed by PCR genotyping using parasite-infected erythrocytes, and the resulting transgenic parasite lines were designated Sel1::AGIA, CA::AGIA, CRMP4::AGIA, and Pb13634::AGIA (**Supplementary Figure S4B**). These transgenic parasite populations were used for further expression and localization analyses.

**Figure 3:**
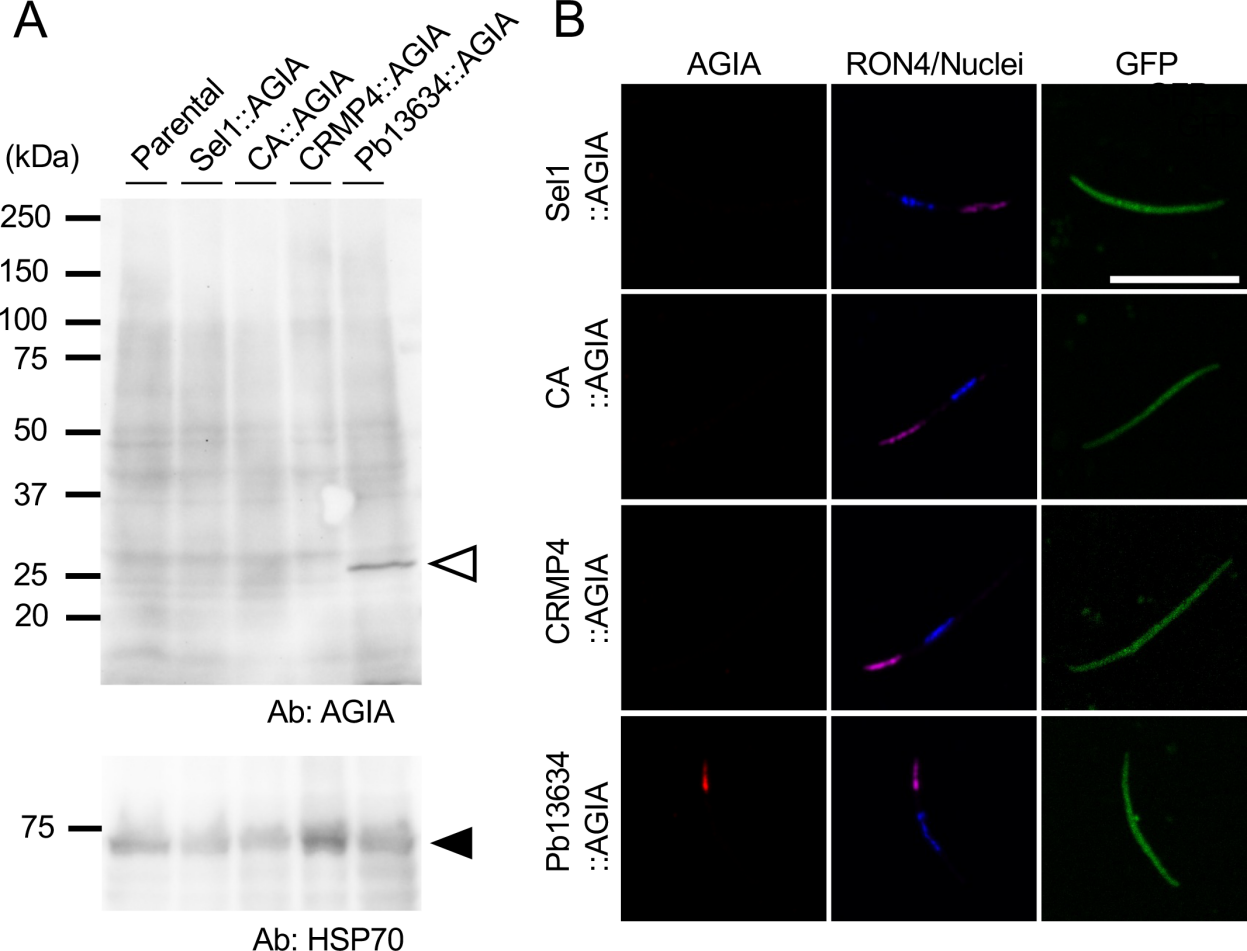
Expression and localization of candidate proteins in oocyst-derived sporozoites. **(A)** (Left panel) Western blotting of 5x10^5^ sporozoites collected from infected mosquito midguts using rabbit anti-AGIA antibodies (1:1,000 dilution) under reducing conditions to detect four AGIA-tagged candidate proteins. An open arrowhead indicates a specific band at 26 kDa, corresponding to the expected size of Pb13634::AGIA. (Lower panel) The same PVDF membrane was incubated with anti-HSP70 antibodies (1:3,000,000 dilution) to demonstrate parasite protein loading, indicated by a closed arrowhead. The sizes of the molecular weight markers are shown on the left. **(B)** Localization of four candidate proteins in sporozoites. IFA was performed using sporozoites isolated from mosquito midguts at day 14 post-feeding, fixed with 4% PFA for 20 minutes, and then incubated with chimeric rabbit-mouse anti-AGIA antibodies (1:1,000 dilution, shown in red) and anti-RON4 antibodies (1:20,000 dilution, magenta) as a rhoptry marker. Parasite nuclei were stained with Hoechst (0.5 µg/ml, blue). GFP expressed in the sporozoite cytosol is shown in green. Scale bar = 10 μm.

To assess protein expression in oocyst-derived sporozoites, mosquito midguts were harvested at day 14 post-feeding on transgenic parasite-infected mice. Protein lysates were electrophoretically separated and subjected to western blotting under reducing conditions using anti-AGIA antibodies. A distinct band corresponding to Pb13634::AGIA was detected at 26 kDa, consistent with its predicted molecular weight (**Figure 3A**), confirming that Pb13634 is expressed in oocyst-derived sporozoites. Despite their high transcript levels in midgut sporozoites and detection by mass spectrometry, the other three candidates could not be detected by western blotting using extracts from 500,000 oocyst-derived sporozoites. IFA using oocyst-derived transgenic sporozoites and anti-AGIA antibodies confirmed that only Pb13634 could be detected in sporozoites (**Figure 3B**). The specific signal corresponding to Pb13634 was colocalized to RON4 at the apical end of sporozoites, suggesting that Pb13634 is expressed in rhoptries. The expression of these candidate proteins was further examined in schizont-enriched lysates by western blotting. In addition to Pb13634, a faint band corresponding to Sel1::AGIA could be detected at its expected size (**Supplementary Figure S5A**).

### 3.6 PBANKA_1363400 is predominantly expressed in sporozoites and localizes to rhoptries

**Figure 4:**
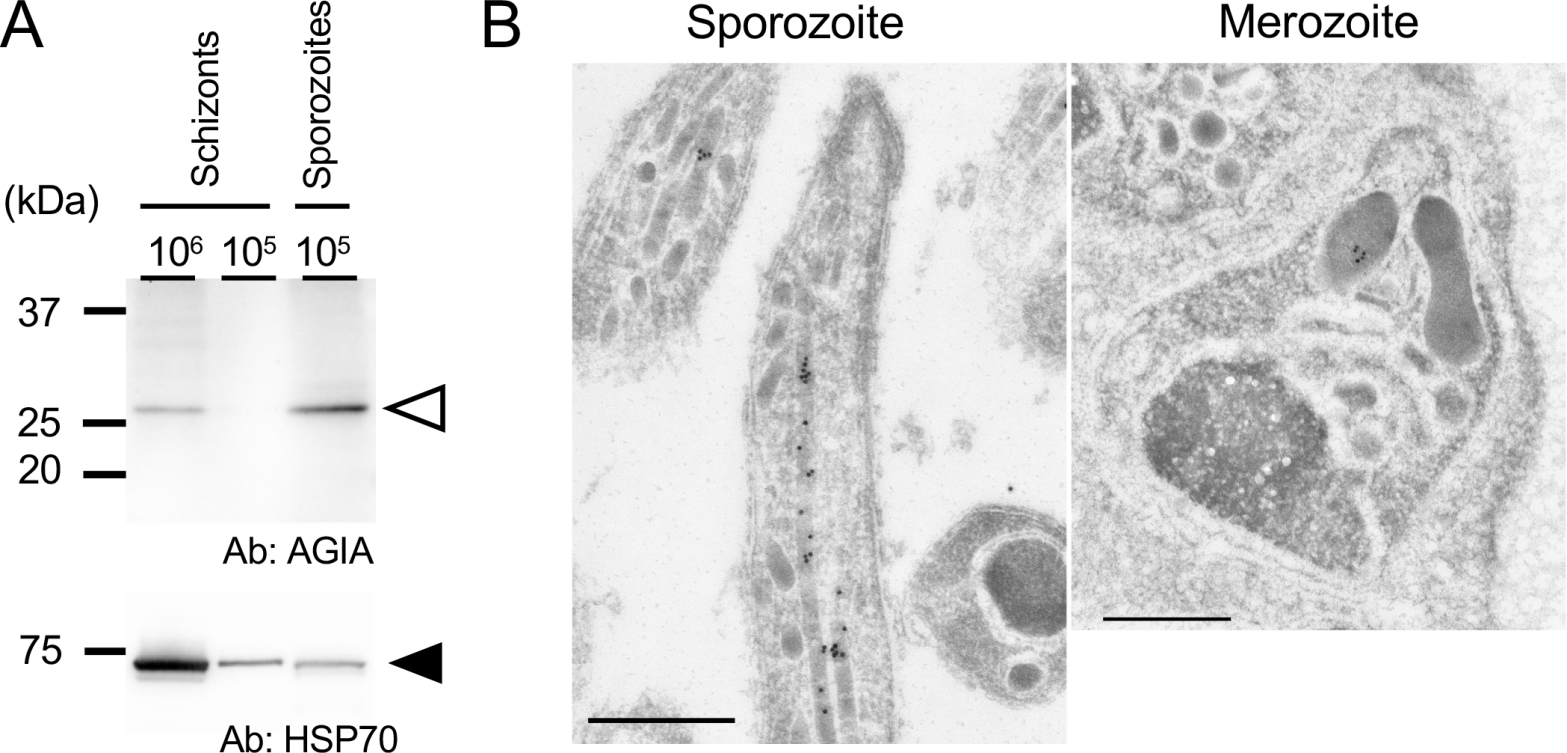
PBANKA_1363400 expression comparison between schizonts and oocyst-derived sporozoites. **(A)** Western blot analysis was performed to compare Pb13634::AGIA protein levels in schizonts and oocyst-derived sporozoites. Lysates from 1x10^6^ and 1x10^5^ schizont enriched parasites were loaded alongside 1x10^5^ oocyst-derived sporozoites. Pb13634::AGIA were detected using rabbit anti-AGIA antibodies (1:1,000 dilution) and specific bands are indicated by an open arrowhead. The Pb13634::AGIA protein amount is higher in oocyst-derived sporozoites compared to the same number of schizonts. (Lower panel) The same PVDF membrane was incubated with anti-HSP70 antibodies (1:1,000,000 dilution) to demonstrate parasite protein loading, as indicated by a closed arrowhead. The sizes of the molecular weight markers are shown on the left. **(B)** Immuno-electron microscopy of Pb13634::AGIA in sporozoites and merozoites. Ultra-thin sections of oocyst-derived sporozoites at day 16 post-feeding and cultured schizonts were stained with rabbit anti-AGIA antibodies (1:100 dilution). Gold particles corresponding to Pb13634::AGIA were detected in rhoptries both in sporozoites and in merozoites formed in schizonts. Scale bars: 500 nm.

The expression profile of Pb13634 was determined using western blot analysis, and demonstrated a significantly higher abundance of Pb13634 in sporozoites compared to a similar number of schizonts (**Figure 4A**). Pb13634 localization was determined by immunoelectron microscopy using transgenic oocyst-derived sporozoites at day 16 post-feeding, as well as *in vitro* cultured schizonts. The results clearly demonstrated that Pb13634 localizes to sporozoite rhoptries, while its localization is limited to the rhoptry bulb in merozoites (**Figure 4B**). IFA revealed that Pb13634 is localized to the apical end of merozoites, while no signals could be detected in gametocytes (**Supplementary Figure S5B**).

In summary, Pb13634 was identified as a novel rhoptry protein expressed predominantly in sporozoites. Pb13634 has an architecture consisting of an N-terminal signal peptide, 248 amino acids, and a predicted molecular weight of 27.5 kDa. BLAST search in VEuPathDB indicates that Pb13634 is conserved among *Plasmodium* spp, but is not present in other apicomplexan genera; and therefore, we named it *Plasmodium* specific rhoptry protein 1 (PRP1).

### 3.7 Disruption of PRP1 was not deleterious to the parasite life cycle

To examine the roles of PRP1 in merozoites and sporozoites, the DiCre system was employed for targeted genome excision (Fernandes et al., 2021; Ramaprasad and Blackman, 2024). A DiCre constitutive expressing line, which could be activated by adding rapamycin, was newly generated by inserting a DiCre expressing cassette into a dispensable intergenic locus using the CRISPR/Cas9 system (**Supplementary Figure S6A**). After confirming the successful integration of the DiCre expression cassette by PCR genotyping (**Supplementary Figure S6B**), the transgenic parasite line was designated GFPAka-DiCre. Then, the CRISPR/Cas9 system was used to introduce two loxP sites into the *prp1* locus, within the 3’UTR and into the coding region with an artificial intron, together with an AGIA-tag at the C-terminus of PRP1 (**Supplementary Figure S7A**; Das et al., 2025; Fernandes et al., 2020, 2022). Successful DNA integration at the expected locus was confirmed by PCR genotyping and the resulting line was named PRP1-2x[loxP] (**Supplementary Figure S7B**). DNA excision at two loxP sites at 16 h after the addition of 10 nM rapamycin during *in vitro* erythrocytic-stage parasite culture was monitored by genotyping PCR and sequencing; and demonstrated that excision occurred with almost 100% efficacy, as no DNA fragment amplification could be detected from non-excision genomic DNA (**Supplementary Figures S7C, D**). Cultured rapamycin treated PRP1-2x[loxP] parasites were inoculated intravenously into naive mice, and the emerged blood stage parasites were stored as ΔPRP1 parasites.

**Figure 5:**
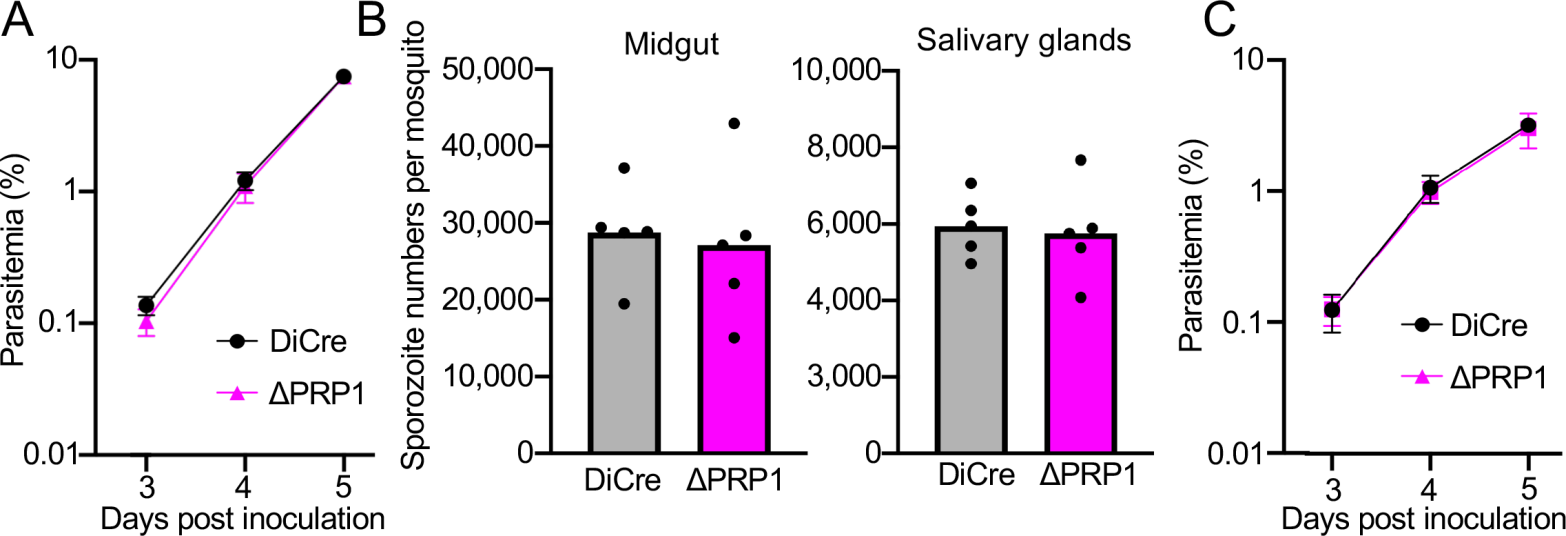
Effect of PRP1 disruption in merozoites and sporozoites. **(A)** The effect of PRP1 disruption on parasite growth in the erythrocytic stage. Blood collected from a PRP1-2x[loxP]-infected mouse was cultured with 10 nM rapamycin for 16 h to obtain ΔPRP1 parasites by recombination. Five female ICR mice were intravenously injected with 20,000 erythrocytes infected with either GFPAka-DiCre (DiCre) or ΔPRP1 parasites and their parasitemias were monitored daily using Giemsa-stained blood smears. Each dot indicates the mean parasitemias from 5 mice, with the standard deviations as bars. Daily parasitemias of ΔPRP1 parasites were not significantly different from those of DiCre using a Mann-Whitney *U* test **(B)** Effect of PRP1 disruption on sporozoite formation and invasion of salivary glands. The mean numbers of sporozoites collected from midguts and salivary glands at day 20 to 23 post-feeding of DiCre or ΔPRP1 infected mosquitoes are shown as bars from four independent feeding experiments and individual numbers are shown as dots. Statistical analyses showed no significant difference in sporozoite numbers between the two parasite lines (Mann-Whitney *U* test). **(C)** The effect of PRP1 disruption on sporozoite infection of mice. Four female C57BL/6 mice were intravenously injected with 15,000 DiCre or ΔPRP1 sporozoites obtained from salivary glands at day 23 post-feeding. Parasitemias of infected mice were monitored daily using Giemsa-stained blood smears. Each dot indicates the mean parasitemias from 4 mice with the standard deviation as bars. Statistical analysis revealed no significant difference in parasitemias at each day between two parasite lines (Mann-Whitney *U* test), demonstrating that PRP1 is dispensable for sporozoite infection of mice.

To investigate possible roles of PRP1 during asexual-stage parasite proliferation, 20,000 ΔPRP1 or GFPAka-DiCre infected erythrocytes were inoculated intravenously into four female ICR mice. Daily monitoring of parasitemias indicated that PRP1 disruption did not affect merozoite infection of erythrocytes nor proliferation (**Figure 5A**). Possible PRP1 roles in sporozoites during invasion of mosquito salivary glands and infection of mouse liver were then assessed, as some rhoptry proteins are crucial for these processes (Baba et al., 2023; Bantuchai et al., 2019; Ishino et al., 2019; Nozaki et al., 2020). Female *A. stephensi* mosquitoes were fed on either ΔPRP1 or GFPAka-DiCre parasite infected mice, and sporozoites were collected from midguts and salivary glands at day 20 to 23 post-feeding. The numbers of ΔPRP1 sporozoites collected from the midguts and salivary glands were equivalent to those of the control, indicating that PRP1 is dispensable for sporozoite formation, release into the hemocoel, and the invasion of salivary glands (**Figure 5B**). The effect of PRP1 disruption on sporozoite infectivity of mice was examined by intravenous inoculation of 15,000 salivary gland-residing sporozoites into four female C57BL/6 mice. The subsequent blood stage parasitemias resulting from ΔPRP1 sporozoite inoculation were not significantly different from those in mice inoculated with control sporozoites (**Figure 5C**). These results demonstrate that the novel *Plasmodium* rhoptry protein, PRP1, is not essential for merozoite invasion of erythrocytes, nor sporozoite invasion of salivary glands or hepatocytes.

## 4 Discussion

### 4.1 Successful biotinylation of known rhoptry proteins using RON12::ultraID expression in *Plasmodium berghei* transgenic sporozoites

In this study we employed proximity-dependent biotin identification to screen for novel rhoptry proteins predominantly expressed in *Plasmodium* sporozoites. To achieve this, we generated a transgenic rodent malaria parasite, *P. berghei*, which expresses RON12::ultraID under its native promoter. RON12 was selected as a bait protein due to its highest expression among known rhoptry molecules in oocyst-derived sporozoites. We demonstrated efficient biotin delivery to developing sporozoites within midgut oocysts by providing mosquitoes with 5% sucrose containing 10 mM biotin for three days starting from day 11 post-feeding, when sporogony occurs inside oocysts (Smith and Barillas-Mury, 2016). This enabled the biotinylation of several proteins in RON12::ultraID expressing sporozoites (**Figure 2A**). Mass spectrometry analysis successfully detected 13 known rhoptry proteins, including RON12 itself. These results highlight the use of UltraID and proximity-dependent biotin identification as a powerful strategy for identifying novel organelle-specific proteins, with a sensitivity sufficient for scarce samples such as *Plasmodium* sporozoites.

### 4.2 Identification of a novel *Plasmodium*-specific rhoptry protein, PRP1, in sporozoites

Previous research demonstrated that most rhoptry molecules found in sporozoites have abundant transcript levels in oocyst-derived sporozoites which are then significantly reduced in salivary gland sporozoites, despite the proteins remaining in rhoptries after sporozoite invasion of salivary glands (Tokunaga et al., 2019). Therefore, to select candidates for novel rhoptry proteins based on transcript patterns, we constructed a comprehensive RNA-seq dataset, consisting of schizonts, oocyst-derived sporozoites at day 17 post-feeding, and salivary gland sporozoites at day 21 post-feeding on mice harboring *P. berghei* ANKA strain parasites. Our dataset confirmed that *ron12* exhibited the highest transcript levels among known rhoptry molecules in oocyst-derived sporozoites (**Supplementary Figure S1**; **Supplementary Table S1**), whereas *rap1* transcription is the highest in schizonts.

Four predicted rhoptry secretory proteins were selected for characterization with AGIA tagging at their C-terminus. Among them, only PBANKA_1363400, herein named *Plasmodium* specific Rhoptry Protein 1 (PRP1), was clearly detected by western blotting in oocyst-derived sporozoites (**Figure 3A**). The low detection efficiency for the other three candidates may be due to low reactivity of AGIA antibodies on linear antigens or processing at their C-terminus, which includes the AGIA tag. Presumably this is due to lower protein amounts of these three candidates in sporozoites, as indicated by proteome analysis using oocyst-derived sporozoites of *P. falciparum* or *P. yoelii* (Lindner et al., 2019). The orthologue of PbPRP1 was listed as 13^th^ or 17^th^ rank in terms of protein amounts in oocyst-derived sporozoites of *P. falciparum* and *P. yoelii*, respectively. The other orthologues in *P. yoelii* ranked from 77^th^ to 1546^th^ out of 1760 proteins detected in oocyst-derived sporozoites. Immuno-electron microscopy clearly demonstrated that PRP1 localizes to rhoptries in sporozoites (**Figure 4B**). Localization of PRP1 in merozoites was restricted to the rhoptry bulb (**Figure 4B**). It has been reported that rhoptry neck proteins, such as RON2, RON4, and RON5, tend to be conserved across species, while many species-specific rhoptry proteins, such as RAMA, localize to the rhoptry bulb in schizonts (Lebrun et al., 2020). Given that PRP1 is conserved in *Plasmodium* species, its localization to the rhoptry bulb in merozoites is consistent with this pattern. Most rhoptry proteins are present throughout the rhoptry organelle in sporozoites (Oda-Yokouchi et al., 2019; Tokunaga et al., 2019), and thus it is reasonable that a similar localization in sporozoites occurs for PRP1 and RON12. As discussed above, the generation of specific antibodies against the other three candidates could clarify their expression and precise localization in sporozoites.

### 4.3 PRP1 is predominantly expressed in oocyst-derived sporozoites

Semi-quantitative western blotting revealed that PRP1 is predominantly expressed in sporozoites. A study by Tokunaga et al. (2019) compared the expression of 12 rhoptry proteins between schizonts and oocyst-derived sporozoites and demonstrated that only the levels of Apical Sushi protein was slightly higher in sporozoites. This unique expression profile distinguishes PRP1 as a novel rhoptry protein that is predominantly expressed in *Plasmodium* sporozoites.

### 4.4 PRP1 is dispensable for either merozoite invasion or sporozoite invasion of target cells

The sporozoite rhoptry proteins RON2, RON4, RON5, and RON11 have been demonstrated to be crucial for both the invasion of mosquito salivary glands and the subsequent infection of mammalian hepatocytes (Baba et al., 2023; Bantuchai et al., 2019; Ishino et al., 2019; Nozaki et al., 2020). Most rhoptry proteins have been shown to be essential for merozoite infection, because parasites could not be isolated which harbor disrupted targeted genes (Ramaprasad and Blackman, 2024). Although *prp1* transcript levels are low in schizonts - approximately 4-fold less than those of *ron2* and *ron4* - we generated a transgenic parasite line in which the *prp1* locus could be conditionally excised upon rapamycin treatment. Following successful insertion of two loxP sites in the *prp1* locus, a portion of the PRP1 coding region was efficiently excised by 10 nM rapamycin addition for 16 h during *in vitro* parasite culture. PRP1 disrupted parasites proliferated normally during blood stage infections, demonstrating that PRP1 is not essential for merozoite invasion of erythrocytes and intraerythrocytic development. Despite its high transcript levels in oocyst-derived sporozoites, PRP1 disrupted sporozoites accumulated normally in mosquito salivary glands and were capable of infecting mice. These results demonstrated that PRP1 is dispensable for both merozoite and sporozoite invasion of target cells, similar to RON12 (Oda-Yokouchi et al., 2019). It is possible that other rhoptry protein(s) may compensate for PRP1 roles. Further investigations, such as RNA-seq analysis in ΔPRP1 sporozoites, could reveal PRP1 roles, particularly its co-functional relationships with other rhoptry proteins. Future attempts at comprehensive identification of sporozoite rhoptry proteins might utilize ultraID baits other than RON12. Characterizations of the molecules identified in these studies might help to illuminate the specific molecular mechanisms by which sporozoites efficiently mediate malaria parasite transmission from mosquitoes to mammals.

## Data Availability

The datasets presented in this study can be found in online repositories. The names of the repository/repositories and accession number(s) can be found below: https://www.ddbj.nig.ac.jp/, PRJDB37937.
