# Supplementary figures and images for: Identification of a novel rhoptry protein expressed predominantly in Plasmodium sporozoites
Source: Front Cell Infect Microbiol. 2026 Jan 26;15:1749149. doi: 10.3389/fcimb.2025.1749149 (PMC12883657; doi:10.3389/fcimb.2025.1749149)

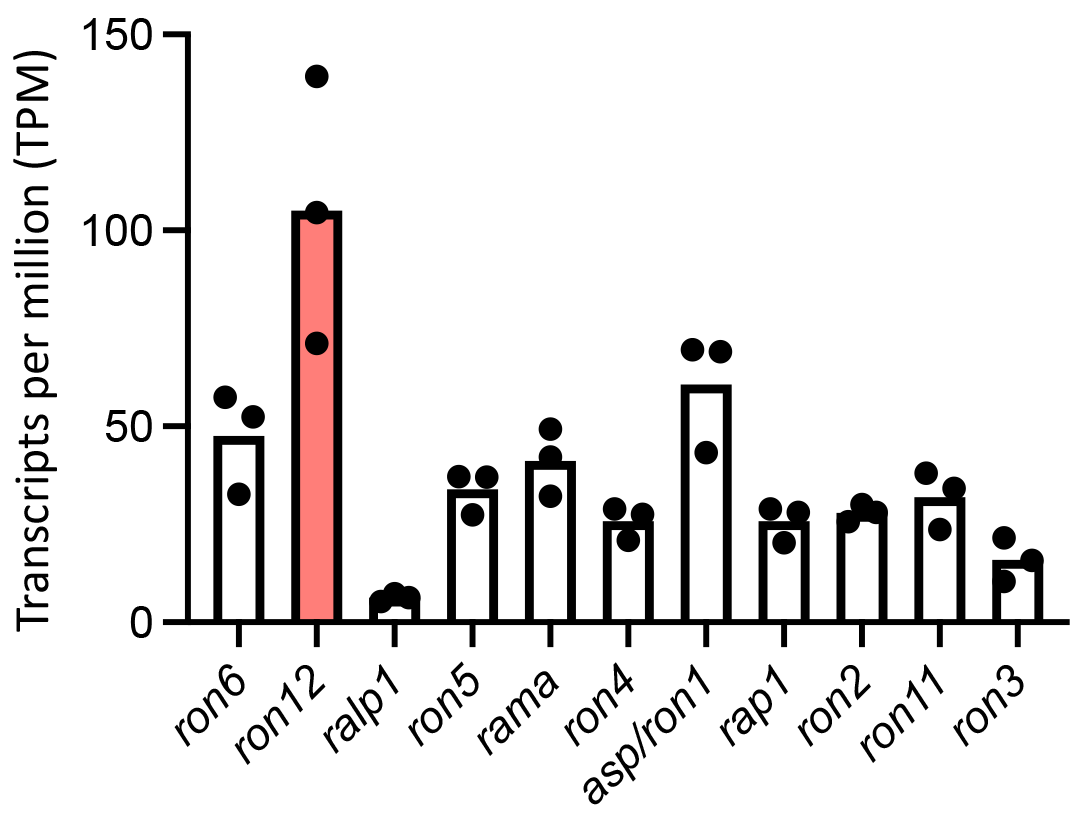

Supplement: Supplementary file 1 [file Image1.tif]

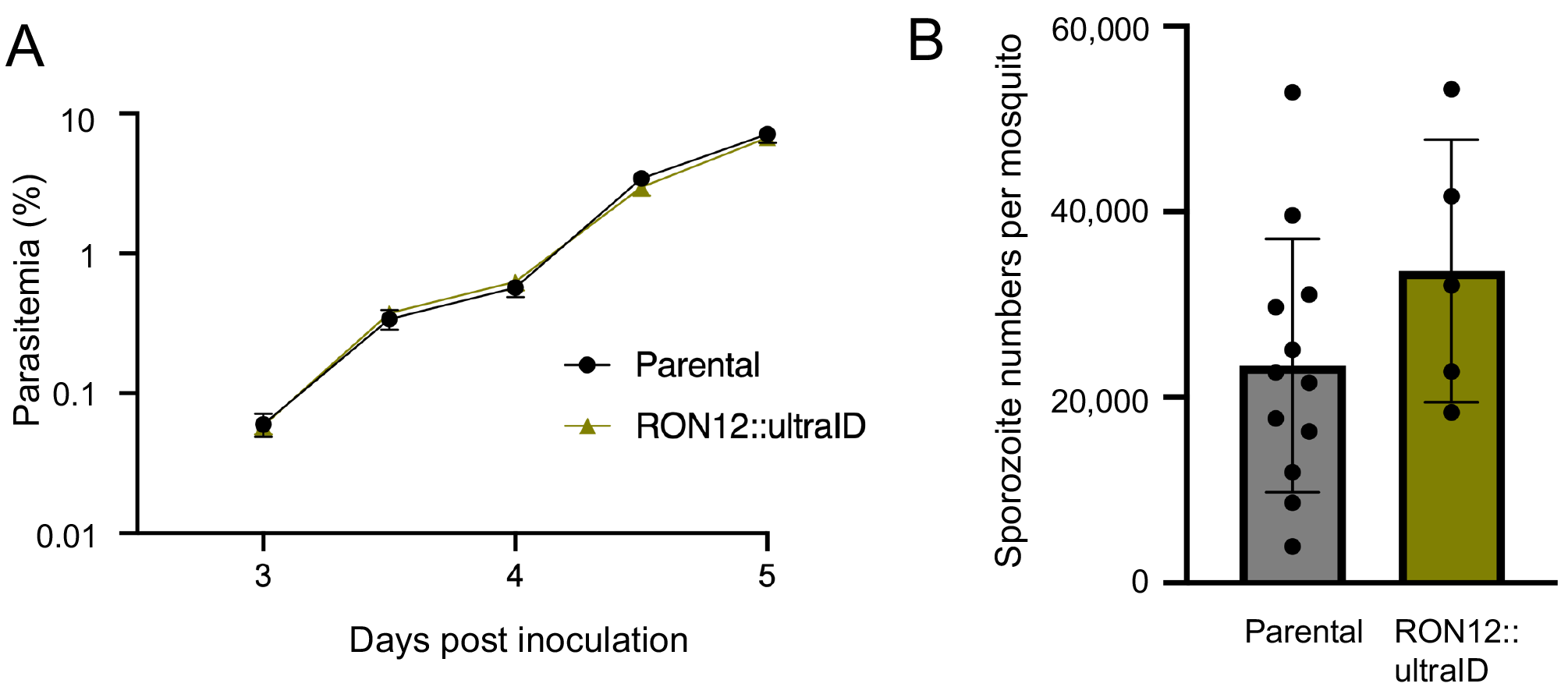

Supplement: Supplementary file 2 [file Image2.tif]

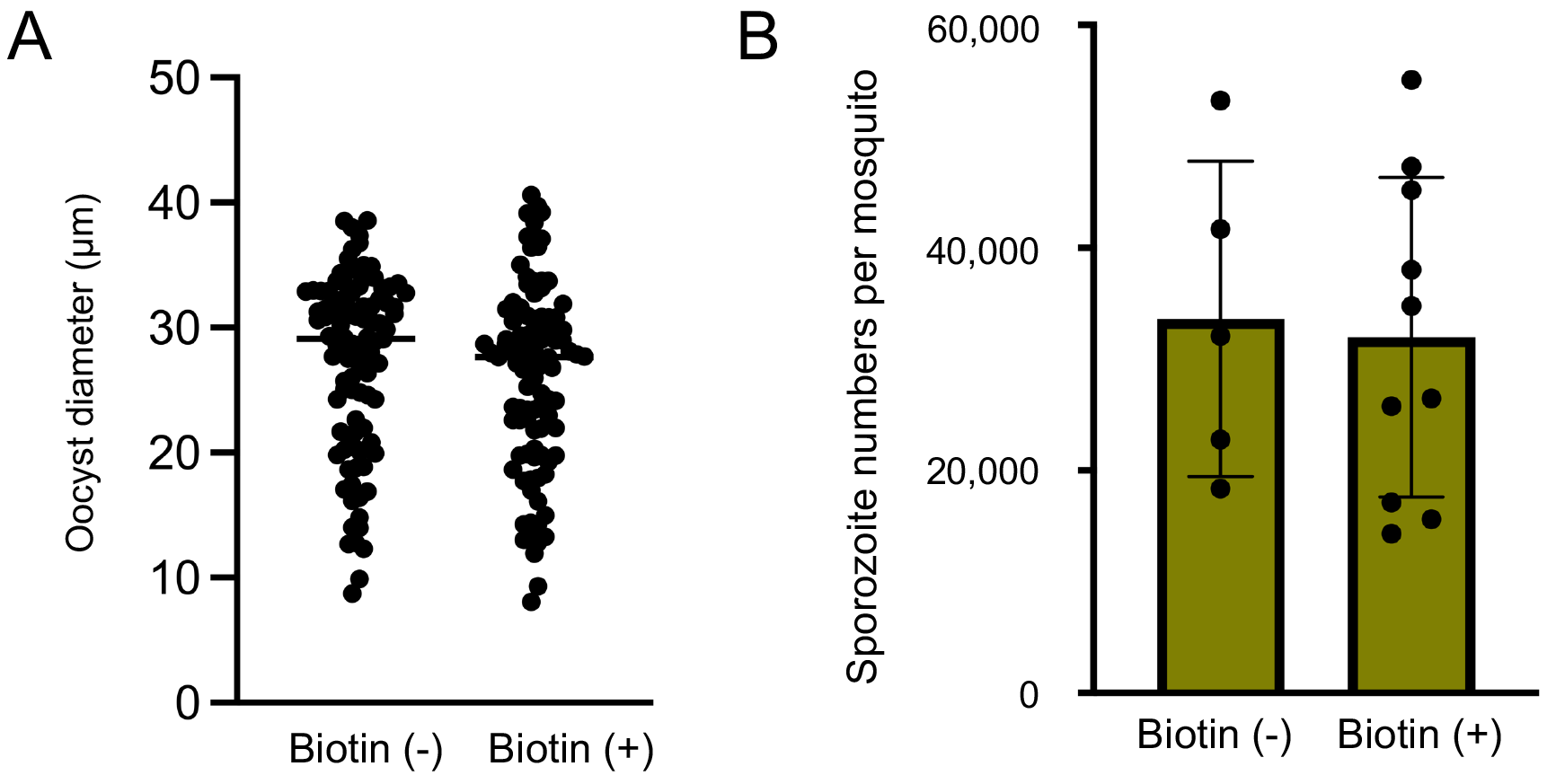

Supplement: Supplementary file 3 [file Image3.tif]

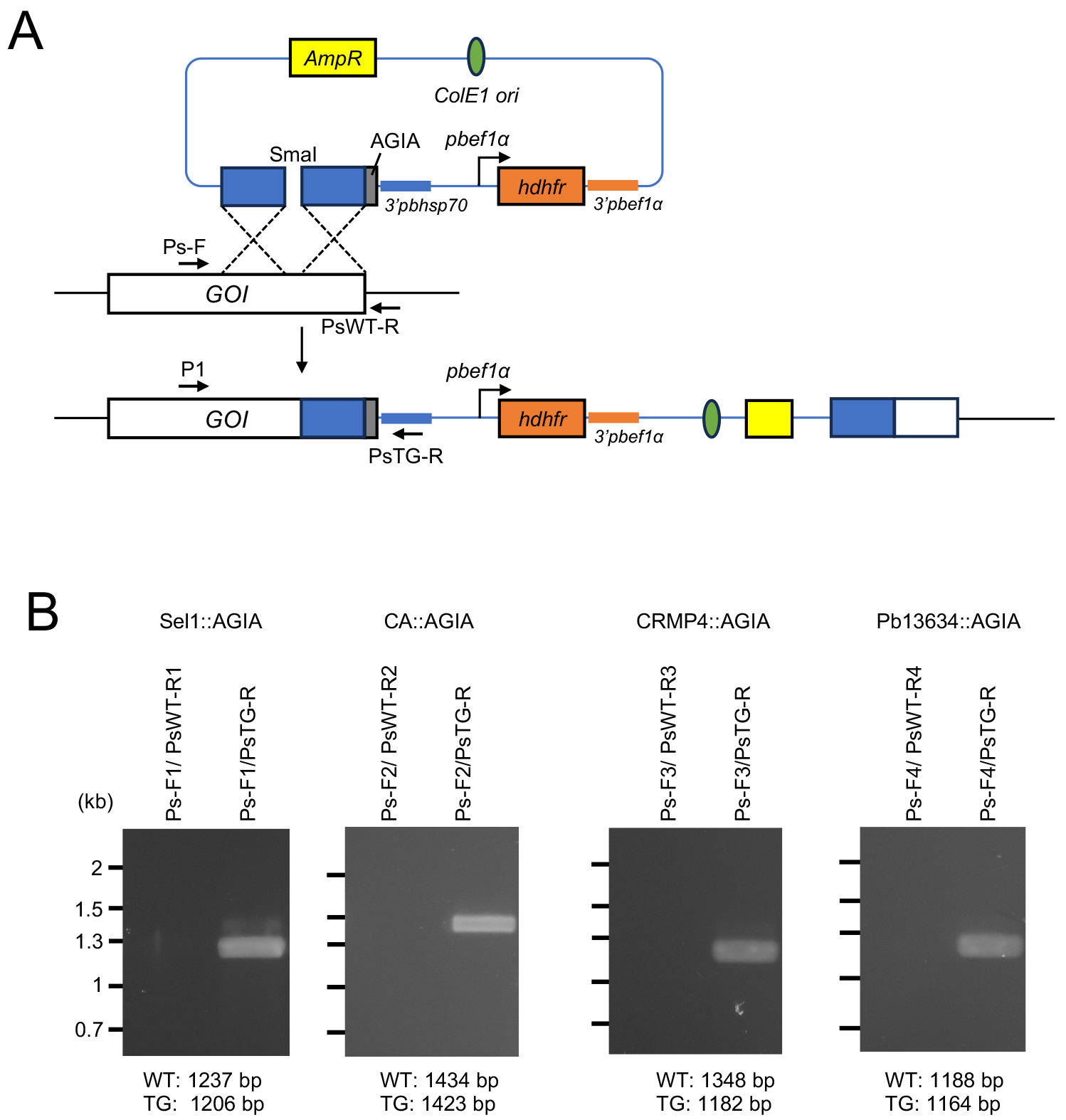

Supplement: Supplementary file 4 [file Image4.tif]

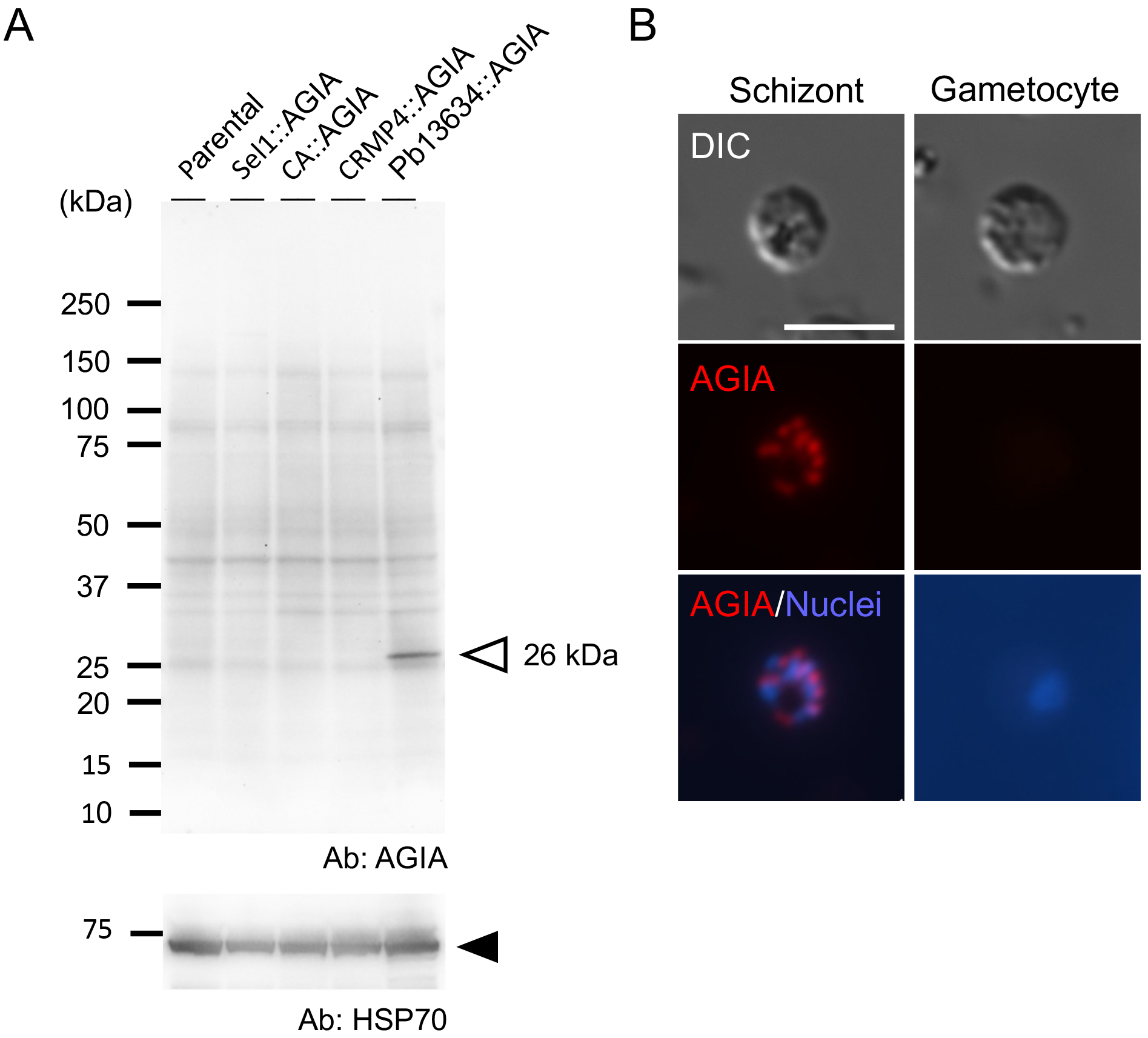

Supplement: Supplementary file 5 [file Image5.tif]

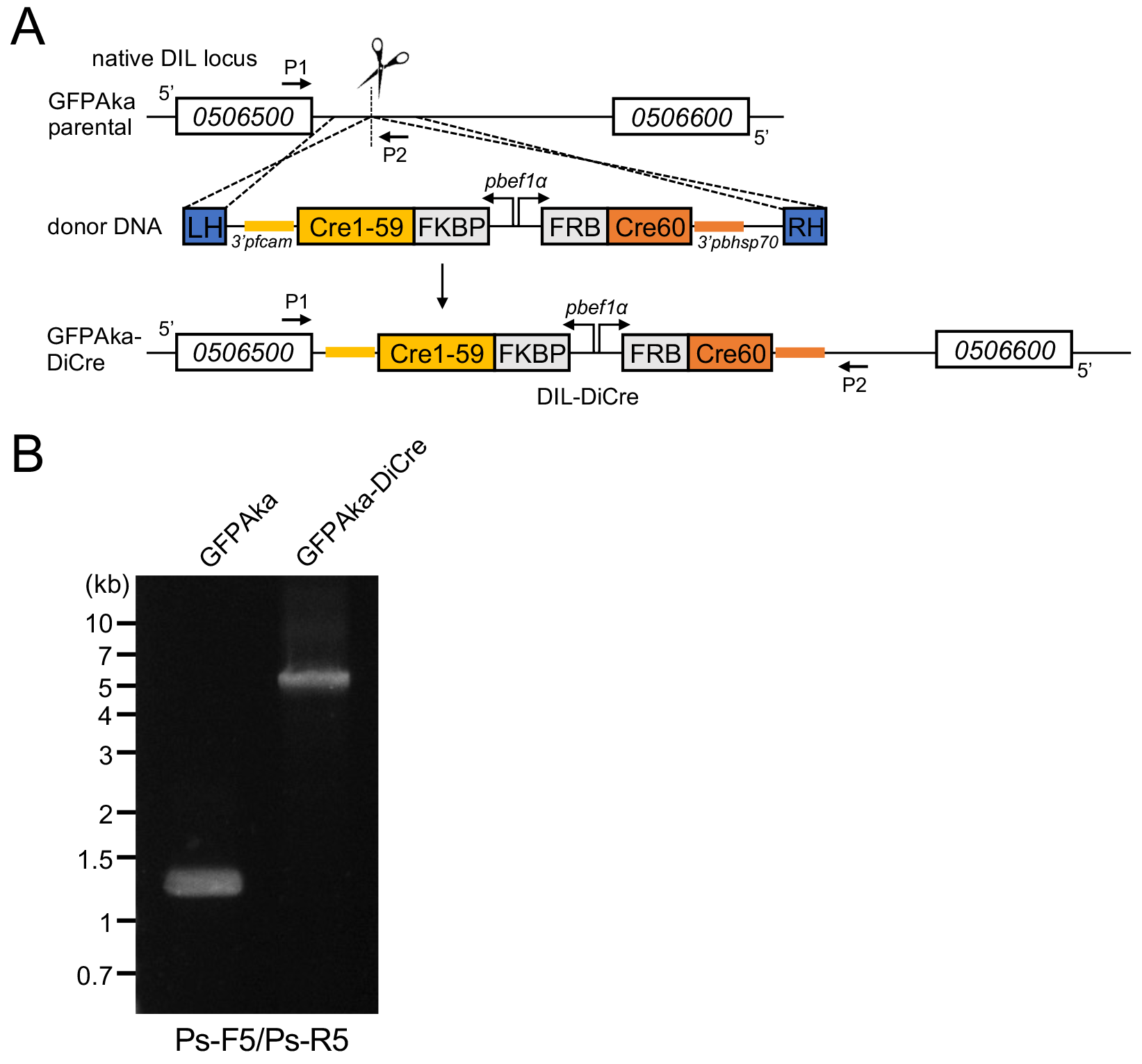

Supplement: Supplementary file 6 [file Image6.tif]

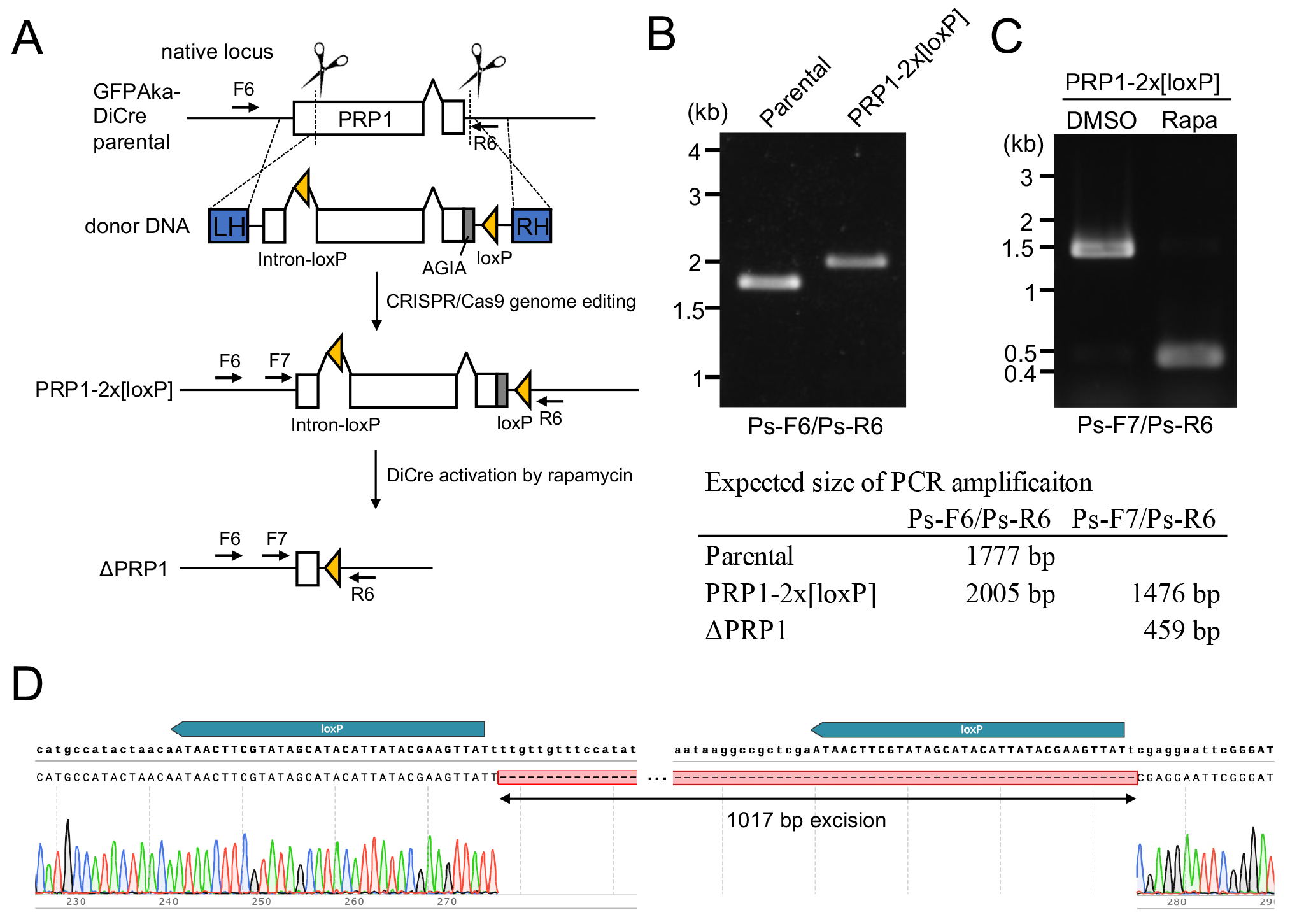

Supplement: Supplementary file 7 [file Image7.tif]
