# Supplementary material for: Identification of a novel rhoptry protein expressed predominantly in Plasmodium sporozoites
Source: Front Cell Infect Microbiol. 2026 Jan 26;15:1749149. doi: 10.3389/fcimb.2025.1749149 (PMC12883657; doi:10.3389/fcimb.2025.1749149)
Supplement: Supplementary file 8 [file DataSheet1.docx]

**Supplementary figure legends**

**Supplementary Figure S1. Transcript comparison among known rhoptry proteins in oocyst-derived sporozoites.**

Rhoptry molecules known to be expressed in sporozoites were selected and their transcripts per million (TPM) levels are shown as dots from RNAseq analysis of three independent oocyst-derived sporozoite mRNA libraries. Average TPMs are shown as bars. The *ron12* transcript level in sporozoites is the highest among the listed rhoptry molecules.

**Supplementary Figure S2. UltraID tagging to RON12 did not affect intraerythrocytic parasite proliferation or sporozoite formation in mosquito midguts.**

(A) The effect of ultraID tagging to RON12 on parasite growth in the erythrocytic stage. Four female ICR mice were intravenously injected with 20,000 erythrocytes infected with either the parental line (GFPAka) or RON12::ultraID parasites and their parasitemias were monitored every 12 h using Giemsa-stained blood smears. Each dot indicates the mean parasitemia from 4 mice, with the standard deviation shown as an error bar. The parasitemias of RON12::ultraID were not significantly different from those of control parasites using the Mann-Whitney *U* test at each time point. (B) The effect of UltraID tagging to RON12 on sporozoite formation in mosquitoes. The mean numbers of sporozoites collected from midguts at day 14 post-feeding of GFPAka or RON12::ultraID infected mosquitoes are shown as bars from at least five independent feeding experiments and individual numbers are shown as dots. No significant different was detected using the Mann-Whitney *U* test.

**Supplementary Figure S3. Biotin treatment of infected mosquitoes did not alter sporozoite formation in oocysts.**

The effect of 10 mM biotin treatment for 3 days from day 11 post-feeding did not alter oocyst size (A) or sporozoite numbers formed in oocysts (B) at day 14. (A) The diameters of oocysts detected by GFP on midguts in GFPAka-infected mosquitoes at day 14 post-feeding are plotted. No significant difference was detected by the Mann-Whitney *U* test between mosquito groups with (+) and without (-) 10 mM biotin treatment from day 11 to day 14 post-feeding. (B) The mean numbers of sporozoites collected from midguts at day 14 post-feeding of RON12::ultraID infected mosquitoes with (+) or without (-) biotin administration are shown as bars from at least five independent feeding experiments and individual numbers are shown as dots. No significant different was detected using the Mann-Whitney *U* test.

**Supplementary Figure S4. Generation of transgenic parasites expressing candidate proteins fused with AGIA-tag at their C-terminus.**

(A) Schematic representation of the insertion of an AGIA-tag at the C-terminal of each rhoptry candidate protein by single-crossover homologous recombination. Recombinant plasmids contain the C-terminus coding sequence (approximately 1.5 kbp) of each candidate protein, fused with the AGIA-tag sequence, followed by the 3′ UTR sequence of the heat shock protein 70 (3*’pbhsp70*). Each plasmid contains a human dihydrofolate reductase expression cassette (*hdhfr*, shown in orange) driven by the *pbef1α* promoter, as a drug-selectable marker. The plasmids were linearized by Smal restriction enzyme before transfection of the parental parasite line (GFPAka). The positions and directions of primers used for PCR genotyping were shown as arrows. (B) Genotyping PCR was performed using genomic DNA extracted from each cloned transgenic parasite. Using specific primer combinations, the expected DNA insertion events were confirmed.

**Supplementary Figure S5.** **Detection of rhoptry candidate proteins in schizonts.**

(A) Western blotting was performed with 5x10^6^ schizont/gametocyte-enriched parasites after overnight in vitro culture of transgenic parasite infected mouse blood as antigens. Incubation with rabbit anti-AGIA antibodies (1:1,000 dilution) could specifically detect Pb13634::AGIA at its expected size (indicated by an open arrowhead). The same PVDF membrane was incubated with anti-HSP70 antibodies (1:1,000,000 dilution) to demonstrate the parasite protein loading, indicated by a closed arrowhead. The sizes of the molecular weight markers are shown on the left. (B) Indirect immunofluorescent analysis of Pb13634::AGIA in schizonts and gametocytes. Parasite-infected erythrocytes, purified by density-gradient centrifugation after 16h of in vitro culture, were plated onto a 0.5mg/ml Concanavalin A coated glass slide. Samples were fixed with 4% PFA for 20 minutes, followed by permeabilization with 0.1% Triton X-100, and then incubated with rabbit anti-AGIA antibodies (1:500 dilution, shown in red) and Hoechst (0.5 µg/ml, blue). Scale bar = 5 μm.

**Supplementary Figure S6. Generation of DiCre expressing transgenic parasites**

(A) Schematic representation of the generation of DiCre expressing parasites to develop a conditional knockdown system. The expression cassette contains two fragments of Cre recombinase (Cre1-59 and Cre60) controlled by a bidirectional *pbef1α* promoter, followed by the 3’UTR of *pfcalmodulin* and the 3’UTR of *pbhsp70*, respectively. This expression cassette was inserted within the dispensable locus (PbANKA_0506500-0506600, DIL) in *Plasmodium berghei* GFP and AkaLuc expressing line (GFPAka) using CRISPR/Cas9 genome editing. (B) Correct insertion of the DNA fragment was confirmed by genotyping PCR using genomic DNA of cloned transgenic parasites (GFPAka-DiCre) and primer sets indicated in Figure S4A.

**Supplementary Figure S7. Generation of PRP1 conditional knockdown parasites.**

(A) Schematic representation of the generation of PRP1 conditional knockdown parasites using the DiCre recombination system. Two loxP sites were inserted within the *prp1* locus: one into the target locus near the 5' end of the coding sequence with the artificial intron (170 bp) and the other was inserted after the stop codon (indicated by yellow triangles). An AGIA-tag sequence was also inserted at the end of PRP1 coding sequence for detection. After rapamycin treatment, the two fragments of DiCre form a complex and allow recombination between the two loxP sites in the *prp1* locus, resulting in *prp1* depletion as shown in the lower illustration. PCR genotyping to demonstrate correct insertion of two loxP sites (B) and excision after rapamycin treatment (C). Genomic DNA was extracted from transgenic parasites, and also from in vitro culture parasites with and without rapamycin treatment. Primer sets used for genotyping are indicated below the images and Figure S7A, and their expected size of amplified DNA are indicated. Upon treatment with rapamycin, only a shorter fragment could be amplified, demonstrating that excision occurred with high efficacy. (D) DNA recombination at two loxP sites was confirmed by DNA sequencing.

Supplementary Table S1. Transcript level profiles of molecules detected in oocyst-derived sporozoites.

Supplementary Table S2. The list of biotinylated protein in sporozoites by RON12::ultraID

Supplementary Table S3. Oligonucleotide primers used in this study.
